# Supplementary material for: Coordination cages integrated into swelling poly(ionic liquid)s for guest encapsulation and separation
Source: Nat Commun. 2024 May 4;15:3766. doi: 10.1038/s41467-024-48135-1 (PMC11069568; doi:10.1038/s41467-024-48135-1)
Supplement: Supplementary file 1 — Supplementary Information [file 41467_2024_48135_MOESM1_ESM.pdf]

Supplementary Information for

Coordination Cages Integrated into Swelling Poly(Ionic Liquid)s for Guest Encapsulation and Separation

Xiang Zhang, Dawei Zhang,* Chenyang Wei, Dehua Wang,* Roy Lavendomme, Shuo Qi, Yu Zhu, Jingshun Zhang, Yongya Zhang, Jiachen Wang, Lin Xu, En-Qing Gao, Wei Yu, Hai-Bo Yang,* and Mingyuan He*

1. Supplementary Methods 2
1.1 Synthesis and characterization of the $Fe^{II}{}_4L_6$ cage 2
1.2 Synthesis and characterization of PIL 3
1.3 Synthesis and characterization of MOC@PILs 5
2. Swellability and mechanical properties of MOC@PILs 10
2.1 Swellability 10
2.2 Mechanical properties 12
3. Host-guest chemistry of the pure cage 13
3.1 Relative size of guests and cage portals 13
3.2 Binding of norbornadiene 15
3.3 Binding of norbornene 18
3.4 Binding of norbornane 20
3.5 Binding of 7-oxabicycloheptane 22
3.6 Determination of binding constants 24
3.7 Binding of norbornadiene and 7-oxabicycloheptane with the solid-state cage 26
4. Host-guest chemistry of the immobilized cage within MOC@PILs 27
4.1 ^{1}H NMR spectral characterization 27
4.2 Binding kinetics 33
4.3 Binding thermodynamics 35
5. Pollutants removal from water 37
5.1 Procedures and removal efficiency 37
5.2 Regeneration of MOC@PILs 44
6. Purification of organic chemicals 46
7. Supplementary Notes 47
8. Supplementary References 48

1. Supplementary Methods

1.1 Synthesis and characterization of the $Fe^{II}{}_4L_6$ cage

The anionic $Fe^{II}{}_4L_6$ cage was synthesized according to the reported procedures with slight modification.[1] Briefly, 4,4'-diaminobiphenyl-2,2'-disulfonic acid (75%, containing water, 2.00 g, 4.35 mmol), tetramethylammonium hydroxide pentahydrate (1.47 g, 8.06 mmol), 2-formylpyridine (840 µL, 8.84 mmol) and $FeSO_4{\cdot}7H_2O$ (0.83 g, 2.98 mmol) were dissolved in 50 mL deionized water into a 100 mL round-bottomed flask giving rise to a dark purple solution. The round-bottomed flask was sealed and filled with nitrogen by three evacuation/charge cycles. The reaction was stirred at 50 °C for 12 h. Dark violet crystals appeared after slow diffusion of excess acetone into the reaction solution and were collected by filtration (yield, 83%). ^{1}H NMR (D_2O, 298 K, 400 MHz): δ 9.32 (s, 12H), 8.70 (d, J= 7.7 Hz, 12H), 8.40 (t, J= 7.7 Hz, 12H), 7.77 (t, J= 6.3 Hz, 12H), 7.54 (d, J= 5.5 Hz, 12H), 7.12 (bs, 12H), 6.45 (s, 12H), 5.83 (bs, 12H), 3.19 (s, 48H) ppm. ^{13}C NMR (D_2O, 298 K, 101 MHz): 173.3, 155.2, 153.0, 147.3, 140.1, 136.9, 133.2, 129.2, 127.1, 118.9, 118.1, 52.4 ppm.

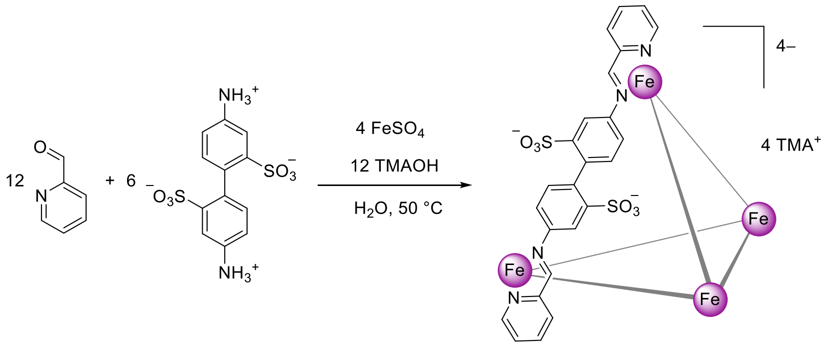

Supplementary Figure 1. Synthesis of the $Fe^{II}{}_4L_6$ cage.

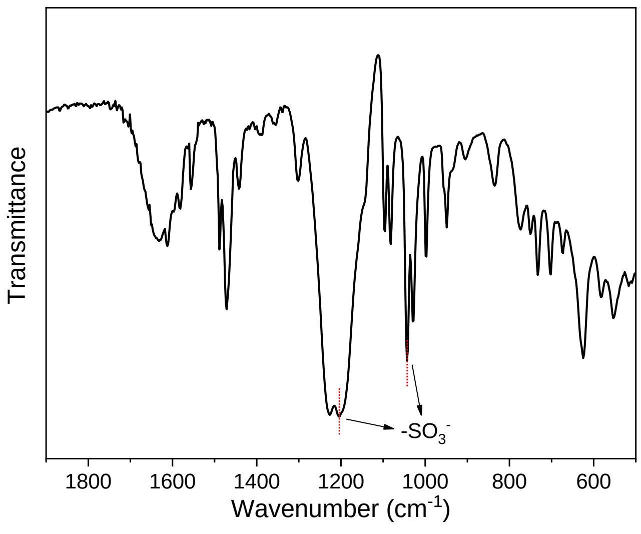

Supplementary Figure 2. FTIR spectrum of the $Fe^{II}{}_4L_6$ cage.

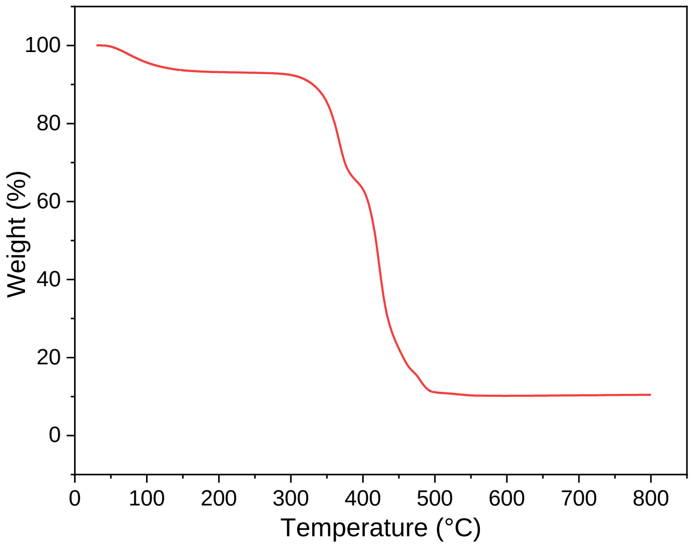

Supplementary Figure 3. Thermogravimetric plot of the $Fe^{II}_4L_6$ cage.

1.2 Synthesis and characterization of PIL

Synthesis of [DVIm]NO_3: The cationic crosslinker **[DVIm]Br** shown in Supplementary Figure 4 was synthesized according to the reported procedures.[2] **[DVIm]Br** (2.32 g, 5 mmol) and $AgNO_3$ (1.70 g, 10 mmol) were dissolved in 20 mL CH_3OH and the solution mixture was stirred at room temperature (rt) for 1 h. White precipitate appeared during stirring, which was removed through filtration. The desired product **[DVIm]NO_3** (1.99 g, 4.6 mmol, 92%) was obtained after evaporation of the filtrate under reduced pressure. ^{1}H NMR (DMSO-d_6, 298 K, 400 MHz): δ 9.41 (s, 2H), 8.19 (s, 2H), 7.84 (s, 2H), 7.32 (dd, J= 15.7, 8.6 Hz, 2H), 5.96 (dd, J= 15.7, 4.2 Hz, 2H), 5.44 (dd, J= 8.3 Hz, 2H), 4.36 (t, J= 4.2 Hz, 4H), 3.77 (t, J= 4.2 Hz, 4H), 3.43 (s, 4H) ppm. ^{13}C NMR (DMSO-d_6, 298 K, 101 MHz): 136.1, 129.3, 124.1, 119.4, 109.3, 69.8, 68.34, 49.6, 40.7, 40.4, 40.2, 40.0, 39.8, 39.6, 39.4 ppm.

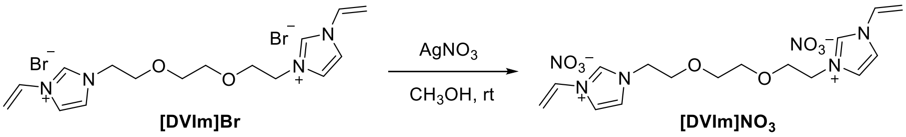

Supplementary Figure 4. Synthesis of **[DVIm]NO_3**.

Synthesis of [VEIM]NO_3: VEIMBr (6.09 g, 30 mmol) and $AgNO_3$ (5.10 g, 30 mmol) were dissolved in 20 mL CH_3OH and the solution mixture was stirred at rt for 1 h. White precipitate appeared during stirring, which was removed through filtration. The desired product **[VEIM]NO_3** (5.17 g, 27.9 mmol, 93%) was obtained after evaporation of the filtrate under reduced pressure. ^{1}H NMR (DMSO-d_6, 298 K, 400 MHz): δ 9.46 (s, 1H), 8.18 (s, 1H), 7.93 (s, 1H), 7.28 (dd, J= 15.6, 8.8 Hz, 1H), 5.94 (dd, J= 15.7, 2.0 Hz, 1H), 5.42 (dd, J= 8.7, 2.0 Hz, 1H), 4.21 (m, 2H), 1.45 (t, J= 7.3 Hz, 3H) ppm. ^{13}C NMR (DMSO-d_6, 298 K, 101 MHz): 135.6, 129.3, 123.4, 119.6, 109.0, 45.1, 40.5, 40.3, 40.1, 39.9, 39.7, 39.5, 39.3, 15.2 ppm.

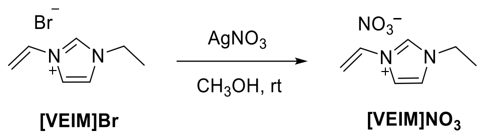

Supplementary Figure 5. Synthesis of **[VEIM]NO_3**.

Synthesis of PIL-NO_3^-: PIL-NO_3^- was synthesized via free radical polymerization. **[DVIm]NO_3** (0.27 g, 0.63 mmol), **[VEIM]NO_3** (4.63 g, 25 mmol) and H_2O (19 mL) were added into a 100 mL round-bottomed flask equipped with a magnetic stirrer. The round-bottomed flask was sealed and filled with nitrogen by three evacuation/charge cycles. Then, 2,2'-azobis[2-methylpropionamidine] dihydrochloride (AIBA) (0.25 g, 5 wt%) was added after stirring the mixture at rt for 0.5 h, and the mixture was stirred at 80 °C for another 24 h. After completion of the polymerization, the polymers were cooled to room temperature and washed with a large amount of water and acetone, and then dried at 60 °C overnight. White powder was obtained (3.28 g, 67%). The swelling capacity of the synthesized PIL-NO_3^- was measured to be 350 based on the method described in Section 2.1.

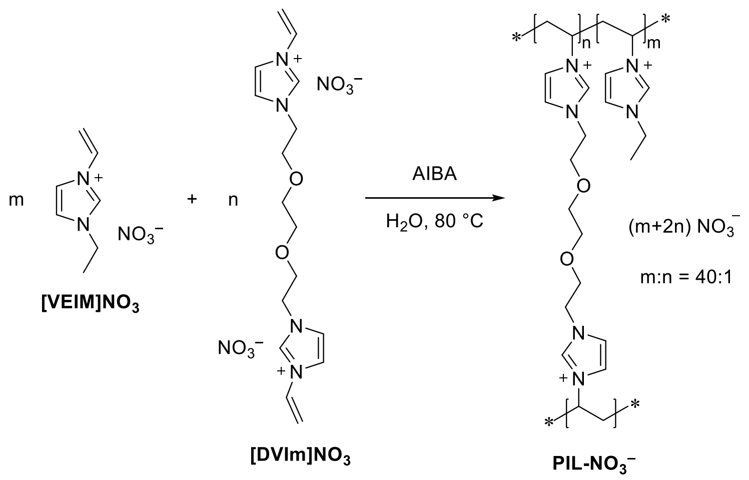

Supplementary Figure 6. Synthesis of the PIL-NO_3^-.

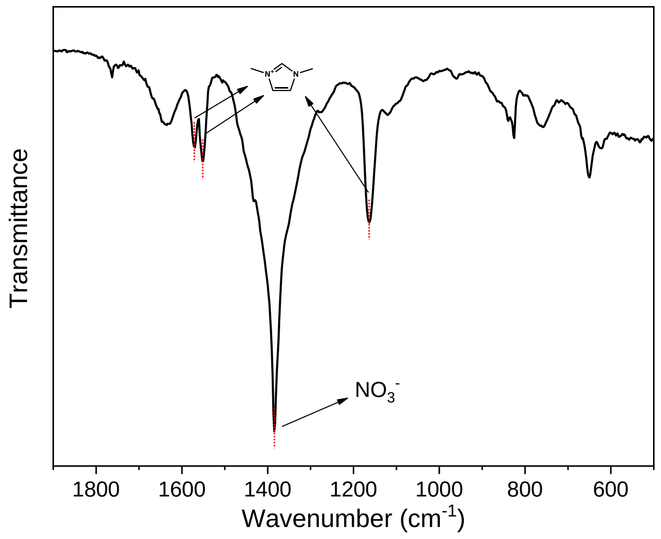

Supplementary Figure 7. FTIR spectrum of the PIL-NO_3^-.

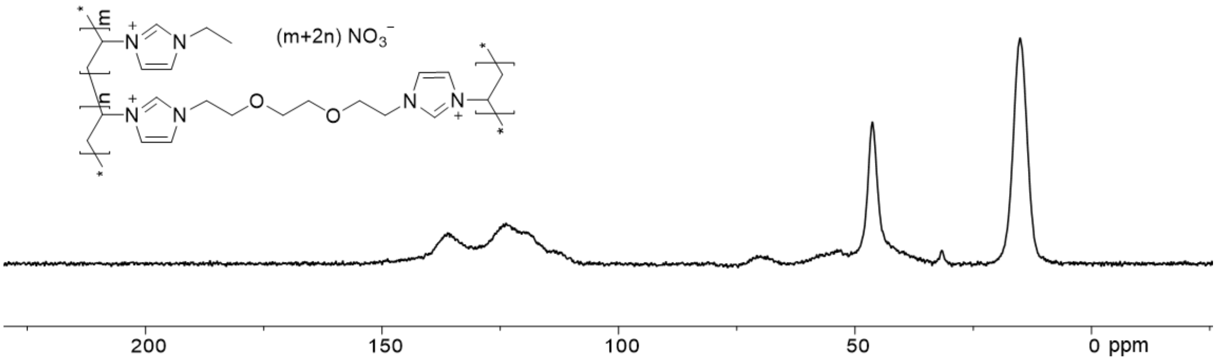

Supplementary Figure 8. Solid-state ^{13}C MAS NMR spectrum of the PIL-NO_3^-.

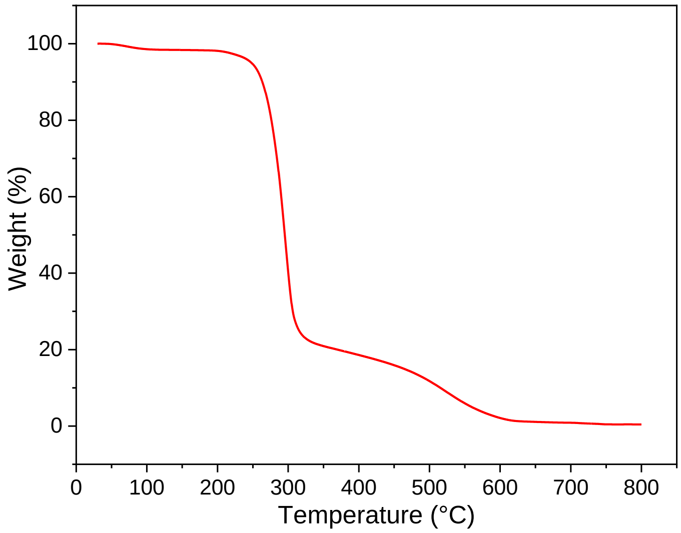

Supplementary Figure 9. Thermogravimetric plot of the PIL-NO_3^-.

Synthesis of PIL-PF_6^-: PIL-PF_6^- was prepared as the control adsorbent for the experiments of pollutant removal from water due to its appropriate swellability (see Section 5). The PIL-NO_3^- (50 mg) was added into 20 mL deionized water and the mixture was equilibrated for 30 min to allow the complete swelling of the PIL. An excess of $NaPF_6$ (54 mg) was added into the solution and the mixture was stirred for 1 h. Centrifugation was conducted to isolate PIL-PF_6^-, which was washed with a large amount of water and then dried at 60 °C overnight. White powder was obtained (62 mg). The swelling capacity of the synthesized PIL-PF_6^- was measured to be 12 based on the method described in Section 2.1, which was lower than that of the pristine PIL-NO_3^- (Q = 350).

1.3 Synthesis and characterization of MOC@PILs

Prior to the synthesis of MOC@PILs, we conducted experiments to confirm the integrity and stability of the $Fe^{II}_4L_6$ cage in the presence of PIL-NO_3^-. After mixing the $Fe^{II}_4L_6$ cage with a large excess of PIL monomers in D_2O (12 equiv. **[VEIM]NO_3** relative to the cage; the ratio between the two is higher than

that of the integrated MOC and the monomer within MOC@PIL **1**), the ^{1}H NMR spectrum of the sample were measured (Supplementary Figure 10). Compared with the ^{1}H NMR spectrum of the $Fe^{II}_4L_6$ cage, no noticeable variation was observed when in the presence of 12 equiv. **[VEIM]NO$_3$**.

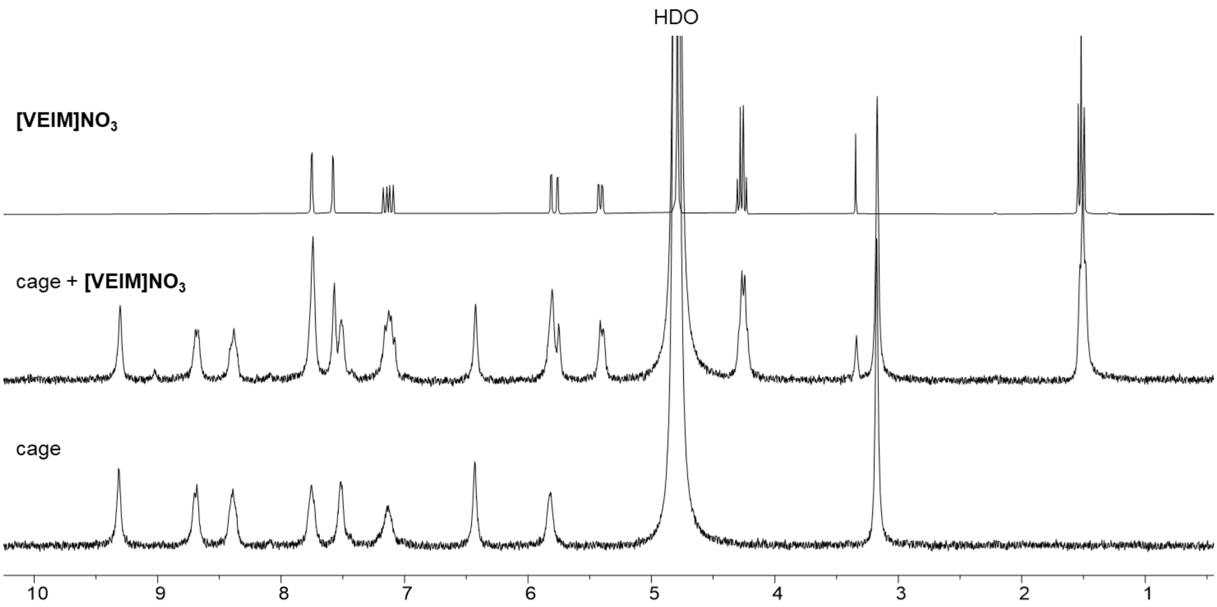

Supplementary Figure 10. ^{1}H NMR (D_2O, 400 MHz, 298 K) spectra of the $Fe^{II}_4L_6$ cage, the $Fe^{II}_4L_6$ cage in the presence of 12 equiv. **[VEIM]NO$_3$**, and **[VEIM]NO$_3$**.

MOC@PILs with differing amounts of the coordination cage were synthesized via an ion exchange method (Supplementary Figure 11). The parent PIL-NO_3^- (0.50 g) was added into 200 mL deionized water. After completely swelling in water, the $Fe^{II}_4L_6$ cage with varying amounts (0.10, 0.18, 0.25, 0.50, 0.75, 1.0, 1.25, and 2.0 g in each) was added into the solution. The mixture was stirred at rt for 1 h. MOC@PILs were obtained by centrifugation, and were washed with a large amount of water. MOC@PILs were deswelled by adding acetone followed by centrifugation. Purple powders (> 0.50 g for each sample) were finally obtained after drying each sample at 60 °C overnight.

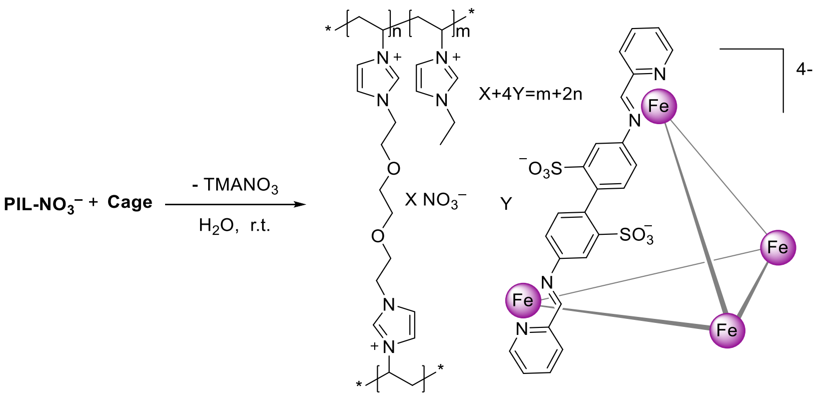

Supplementary Figure 11. Synthesis of MOC@PILs.

The supernatants were monitored using ^1H NMR spectroscopy during the synthesis of MOC@PILs. As nondeuterated water was used during the synthesis, a capillary filled with D_2O was inserted into the NMR tube containing the supernatant sample for field frequency locking (Supplementary Figure 12).

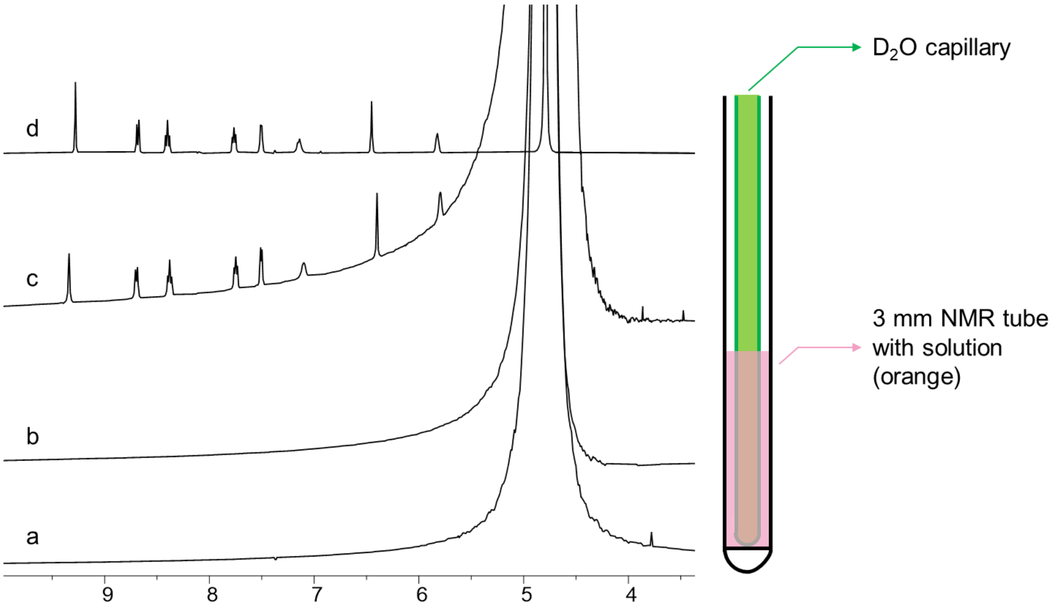

Supplementary Figure 12. ^1H NMR (400 MHz, 298 K) spectra of the supernatants obtained from the complete swelling of 0.5 g PIL-NO_3^- in 200 mL water (a), thorough mixing of 0.5 g PIL-NO_3^- and 1.0 g cage in 200 mL water (b), thorough mixing of 0.5 g PIL-NO_3^- and 2.0 g cage in 200 mL water (c), and the spectrum of the $Fe^{II}_4L_6$ cage (d). A capillary filled with D_2O was inserted into the NMR tube containing the supernatant sample for field frequency locking.

The Fe contents of MOC@PILs were measured by ICP-AES after digesting the samples of MOC@PILs (40 mg) with concentrated nitric acid (2 mL) at 120 °C for 12 h. The amounts of the anionic cage within the eight samples of MOC@PILs were calculated to be 0.11, 0.24, 0.32 (MOC@PIL **1**), 0.42 (MOC@PIL **2**), 0.51 (MOC@PIL **3**), 0.62 (MOC@PIL **4**), 0.65 (MOC@PIL **5**), and 0.74 (MOC@PIL **6**) g/g, respectively, based on the amounts of Fe measured in each sample.

We first used a second ion exchange to prove the successful immobilization of the cage onto the PIL chains (Supplementary Figure 13). The addition of an excess of sodium nitrate into the aqueous solution containing swollen MOC@PILs resulted in the release of the anionic $Fe^{II}_4L_6$ cage into the solution pairing with Na^+. After centrifugation to remove the insoluble polymers, the solution containing the released cage was collected and the corresponding ^1H NMR spectrum was recorded.

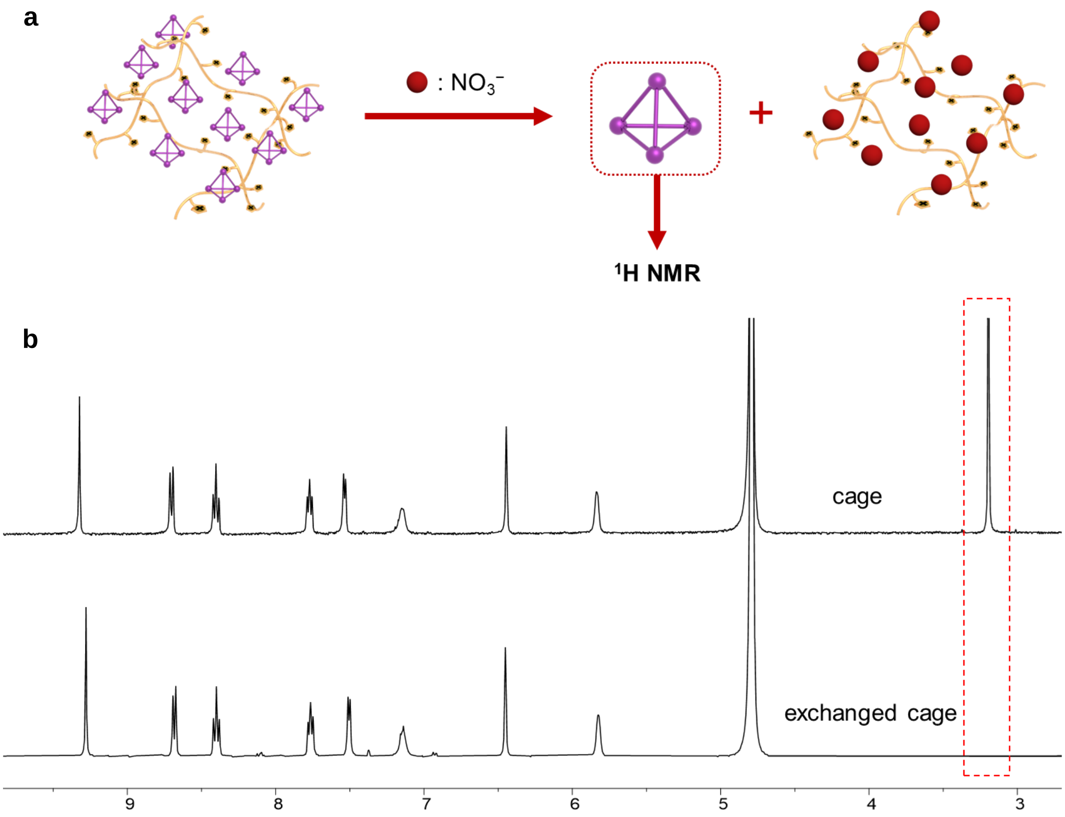

Supplementary Figure 13. (a) Schematic illustration of the anion exchange strategy for proving the existence of the anionic cage within MOC@PILs. (b) ^{1}H NMR (D_2O, 400 MHz, 298 K) spectra of the synthesized anionic cage (TMA^+ as the counterion) and the cage released from MOC@PIL **6** by adding $NaNO_3$.

The FTIR spectra of MOC@PILs show peaks at 1385, 1190, and 1043 cm^{-1}, which can be attributed to the stretching vibration of NO_3^-, and the asymmetrical and symmetrical stretching of $-SO_3^-$, respectively (Supplementary Figure 14).[3] The peaks at 1569, 1550, and 1161 cm^{-1} are attributed to the skeleton stretching vibration of imidazolium ring and the C(2)−H bond bending vibration of the imidazolium group,[4] proving the existence of the PIL structure.

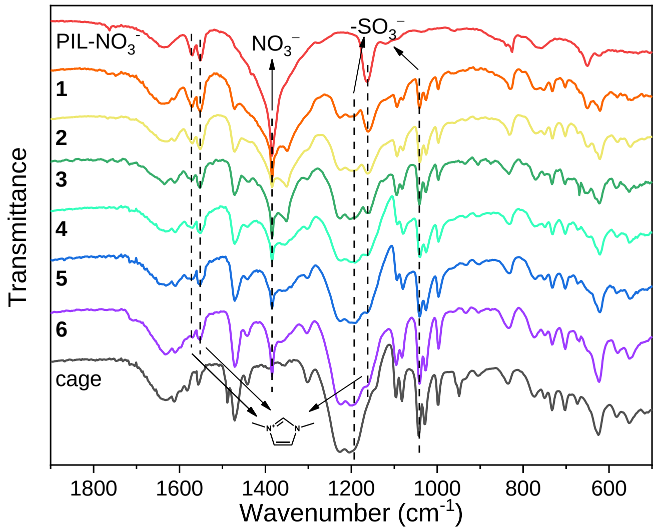

Supplementary Figure 14. FTIR spectra of the parent PIL-NO_3^-, the anionic cage, and MOC@PILs **1-6**.

The solid-state ^{13}C MAS NMR spectrum of MOC@PIL **6** shows that a series of typical broadened signals in the region of 120–180 ppm, including the resolved imine peak at 176 ppm, are presented, corresponding to the signals of the $Fe^{II}_4L_6$ cage (Supplementary Figure 15). The two signals at 15.1 and 46.3 ppm are attributed to the peaks of the ethyl units linked to the imidazolium rings of the PIL.

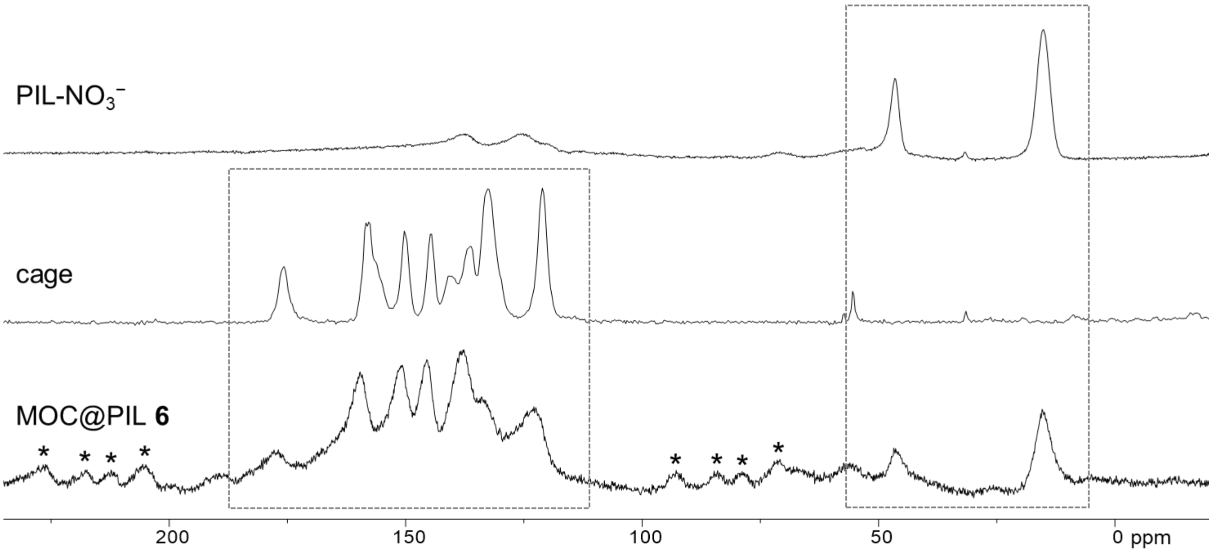

Supplementary Figure 15. Solid-state ^{13}C MAS NMR spectra of the parent PIL-NO_3^-, the anionic cage, and MOC@PIL **6**. The spinning sidebands on the spectrum of MOC@PIL **6** are labelled.

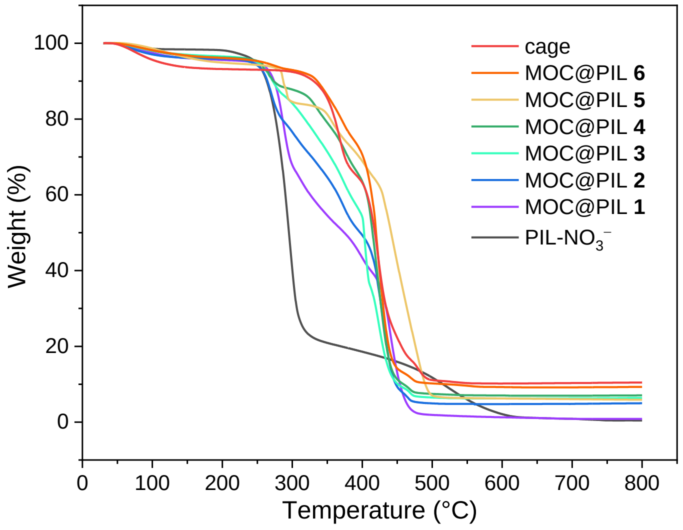

Supplementary Figure 16. Thermogravimetric plots of the parent PIL-NO_3^-, the anionic cage, and MOC@PILs **1-6**.

2. Swellability and mechanical properties of MOC@PILs

2.1 Swellability

The swellability of MOC@PILs were measured as follows: Samples of MOC@PILs (50 mg) were added into sealed bottles containing 30 mL water; The swelling process for each sample was allowed to equilibrate for 12 h at rt; The residue water was then removed through centrifugation and the obtained swollen MOC@PILs were weighed subsequently. The swelling capacity (Q) was calculated according to the following equation:

$$Q = \frac{m_{swollen} - m_{dry}}{m_{dry}} \quad (1)$$

where m_{dry} and $m_{swollen}$ refer to the mass of MOC@PILs before and after complete swelling in water. The swelling capacity were calculated to be 327, 322, 317 (MOC@PIL **1**), 219 (MOC@PIL **2**), 157 (MOC@PIL **3**), 101 (MOC@PIL **4**), 78 (MOC@PIL **5**), and 5 (MOC@PIL **6**), respectively. Moreover, the swollen MOC@PILs could shrink through addition of acetone as unfavorable solvent.

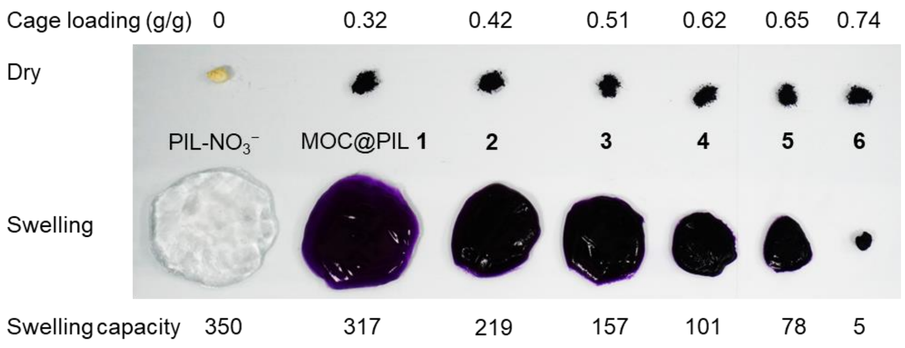

Supplementary Figure 17. Photographs of the swelling behavior of the parent PIL-NO_3^- and MOC@PILs **1-6**.

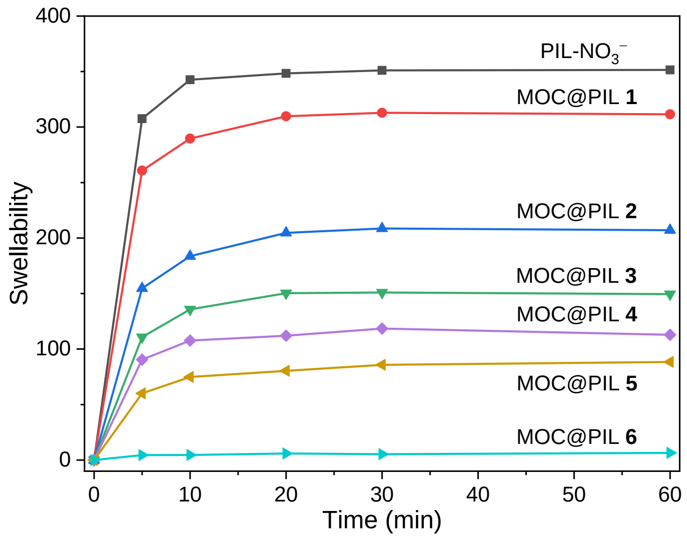

Supplementary Figure 18. Kinetics of swelling of the parent PIL-NO_3^- and MOC@PILs **1-6** in water.

^1H NMR spectra of the fully swollen MOC@PILs **1-6** and the parent PIL-NO_3^- were measured to investigate the electrostatic interactions between the immobilized cages and the PIL chains of MOC@PILs (Supplementary Figure 19). Moreover, for the same purpose, ^1H NMR spectra of MOC@PIL **1** at different swelling degrees (50, 100, 200, 317-fold) by adding differing amounts of water were also measured (Supplementary Figure 20).

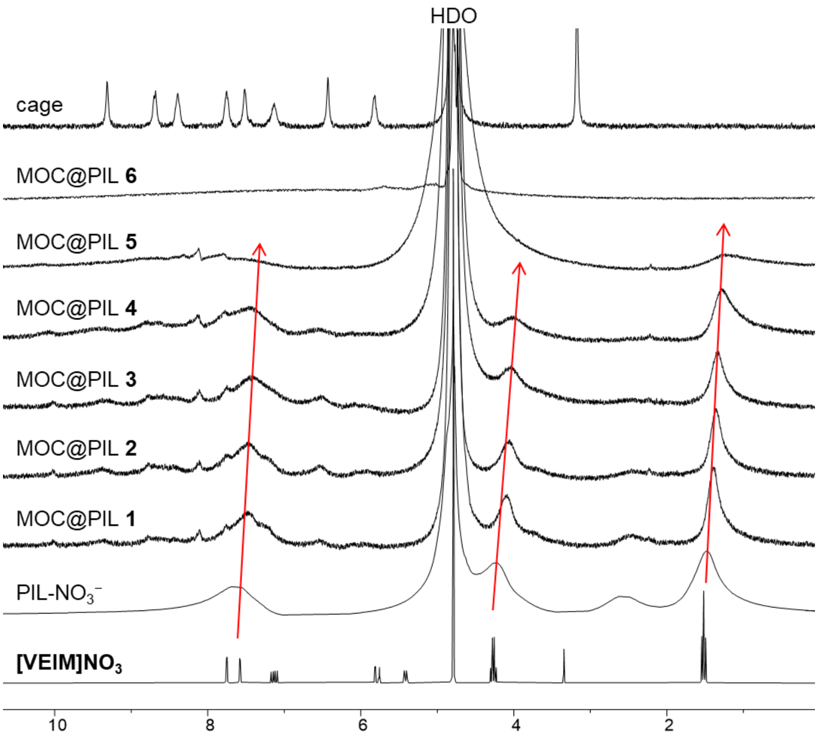

Supplementary Figure 19. ^1H NMR (D_2O, 400 MHz, 298 K) spectra of monomer **[VEIM]NO₃**, the fully swollen gels of PIL-NO_3^- and MOC@PILs **1-6**, and the $Fe^{II}_4L_6$ cage.

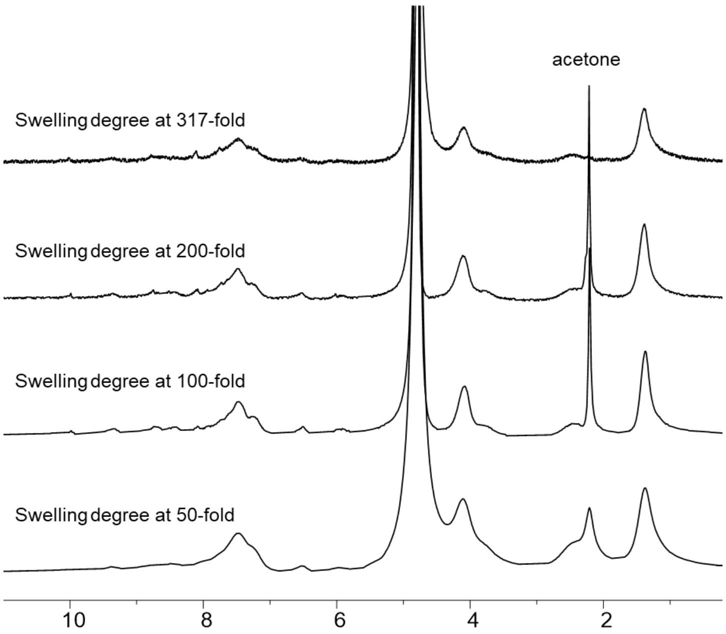

Supplementary Figure 20. ^1H NMR (D_2O, 400 MHz, 298 K) spectra of MOC@PIL **1** at different swelling degrees (50, 100, 200, 317-fold). The peak of acetone originally left within the NMR tubes for cleaning is labelled.

2.2 Mechanical properties

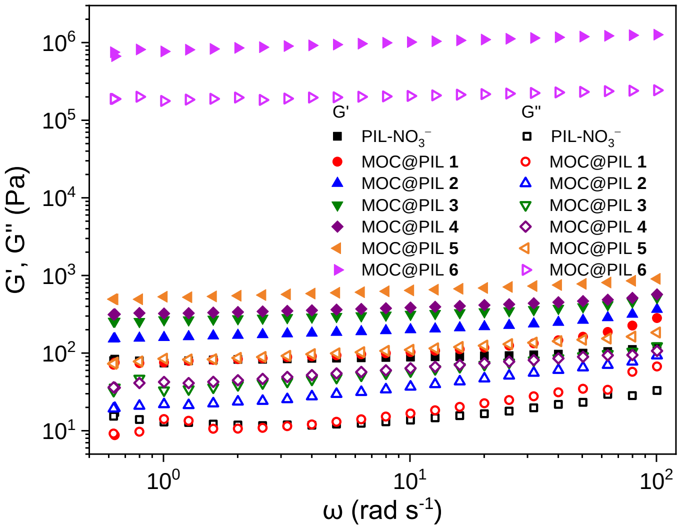

Supplementary Figure 21. Frequency sweeps in oscillatory rheology of the swollen PIL-NO_3^- and MOC@PILs **1-6** ranging from 0.1 to 100 rad s^{-1} at a 1.0% strain amplitude.

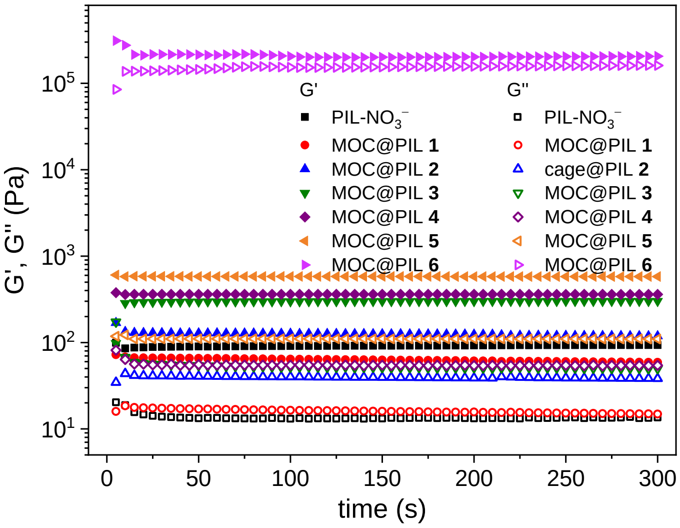

Supplementary Figure 22. Time-dependent oscillatory curves of the swollen PIL-NO_3^- and MOC@PILs **1-6** at a 0.1% strain amplitude.

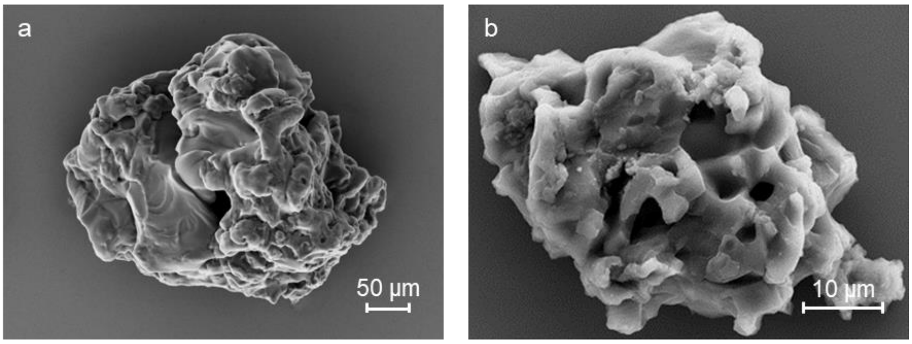

Supplementary Figure 23. SEM images of the agglomerate PIL-NO_3^- (a) and MOC@PIL **4** (b).

3. Host-guest chemistry of the pure cage

The $Fe^{II}_4L_6$ cage was previously reported to bind a set of hydrophobic guests in water through hydrophobic interactions, such as benzene, fluorobenzene, cyclohexane, cyclohexene, dioxane, CH_2Cl_2, and $CHCl_3$ (Supplementary Figure 24).[5] In this work, we explored the binding of four new guests, including norbornane, norbornene, norbornadiene, and 7-oxabicycloheptane. These guests are larger than those previous guests, requiring higher degrees of cage flexibility or even metal-ligand bond reversibility for guest encapsulation. It was found that all of the new guests were bound efficiently by the cage in slow exchange on the NMR time scale at 25 °C. Different from binding those previous smaller guests, the new host-guest mixtures were necessary to equilibrate for a few hours at 50 °C prior to acquisition of NMR spectra. Binding constants were determined through NMR titrations by adding the guests to the cage solution and using *tert*-butanol as the internal standard. The resulting host-guest complexes were characterized by ^{1}H NMR and 2D NMR spectroscopy. In particular, NOESY experiments showed clear NOE correlations between the bound guest signals and occupied cage, demonstrating the internal binding of the guests within the cage.

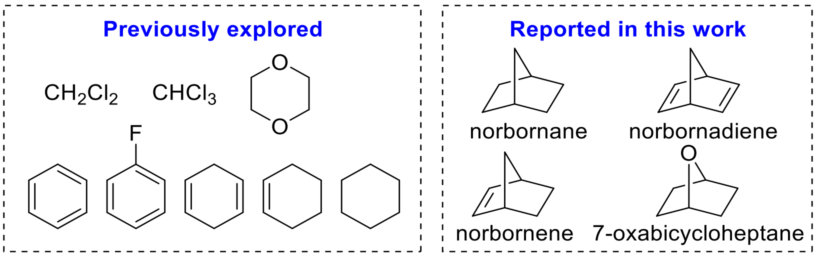

Supplementary Figure 24. Guests investigated in this work. These include a set of previously reported smaller guests and a set of newly explored three-dimensional guests.

3.1 Relative size of guests and cage portals

All guests studied herein are significantly larger than the portal of the static $Fe^{II}_4L_6$ cage from single crystal data (1.7 Å diameter for CCDC 784594, see Supplementary Figure 25). In a dynamic system, the conformational motion of the cage is expected to enlarge its entrance portals thus allowing some small guests (e.g. CH_2Cl_2) to enter the cavity. Such a conformation motion is, however, insufficient to permit the entrance of larger guests. Based on the models, the shortest dimensions of bicyclic guests (5.45–5.97 Å) are longer than those for monocyclic guests (3.54–5.27 Å), while both are significantly larger than the diameter of the cage portal (1.7 Å). Moreover, it was previously shown that the volume and shape, described by their asphericity Ω_A (see Section 7), of guests can be correlated to their inclusion kinetics following the value V/Ω_A (inverse correlation).[5] As listed in Supplementary Table 1, the V/Ω_A parameter of new bicyclic guests studied herein (norbornane derivatives) is one order of magnitude larger than monocyclic cyclohexane derivatives, which is consistent with the much slower inclusion kinetics observed for bicyclic guests. Moreover, the above analysis doesn't take into consideration the conformational flexibility of guests. For instance, the transition between chair and boat

conformations of cyclohexane or 1,4-dioxane could facilitate their insertion in the cage portals. The new bicyclic guests (norbornane derivatives) are larger and conformationally more rigid than monocyclic cyclohexane derivatives which further exacerbates the difference in expected inclusion kinetics.

Note that although the volumes of monocyclic guests are smaller than those of bicyclic guests that may affect the inclusion kinetics, monocyclic guests could rotate to fill the cavity space to have binding affinities close to that of bicyclic "3D" guests.

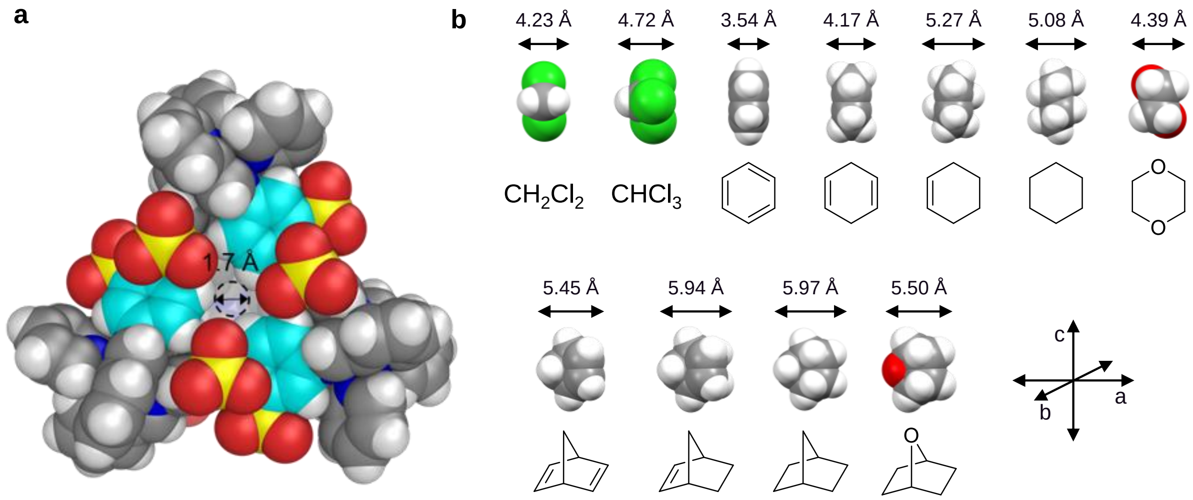

Supplementary Figure 25. X-ray crystal structure of the $Fe^{II}_4L_6$ cage (a) and PM7-optimized molecular models of guests (b). The diameter of the cage portal and the shortest dimension of each guest have been labelled.

Supplementary Table 1. Geometrical features of guest molecules.

**Guests**	**a (Å)**[a]	**b (Å)**[a]	**c (Å)**[a]	**V (Å^3)**[b]	**Asphericity Ω_A**	**V/Ω_A**
CH_2Cl_2	4.23	4.65	6.51	62.27	0.1806	344.72
$CHCl_3$	4.72	6.12	6.50	78.54	0.0546	1438.40
Benzene	3.54	6.70	7.36	88.82	0.0625	1421.06
1,4-Cyclohexadiene	4.17	6.73	6.75	95.28	0.0568	1678.40
Cyclohexene	5.27	6.73	7.19	102.38	0.0452	2267.22
Cyclohexane (chair)	5.08	6.73	7.40	109.25	0.0393	2781.96
1,4-Dioxane (chair)	4.39	5.45	6.37	90.06	0.0427	2111.04
Norbornadiene	5.45	6.16	6.73	103.47	0.00543	19055.69
Norbornene	5.94	6.39	6.72	110.02	0.00551	19984.24
Norbornane	5.97	6.71	6.74	116.66	0.00650	17942.70
7-Oxabicycloheptane	5.50	6.44	6.74	107.22	0.00849	12628.46

[a] a < b < c measured as the shortest orthogonal dimensions (Supplementary Figure 25) of the molecules following atomic radii reported in Section 7.
[b] Molecular volumes calculated with the MoloVol program (see Section 7).

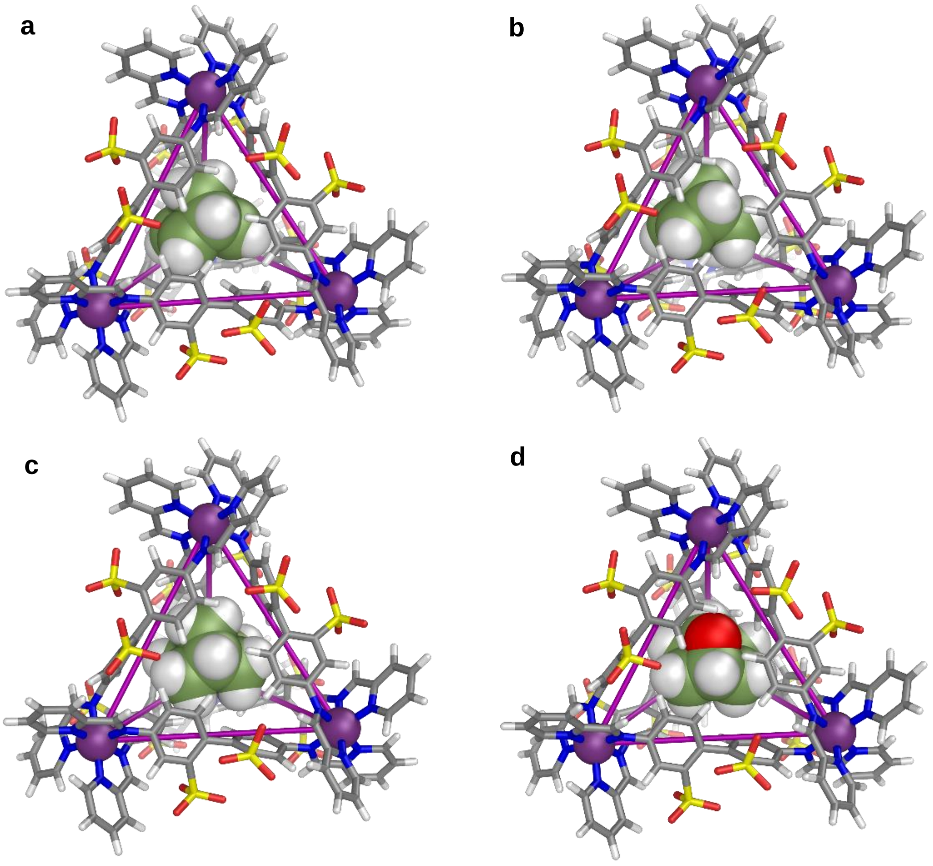

Supplementary Figure 26. PM7-optimized molecular models of norbornadiene⊂MOC (a), norbornene⊂MOC (b), norbornane⊂MOC (c), and 7-oxabicycloheptane⊂MOC (d).

3.2 Binding of norbornadiene

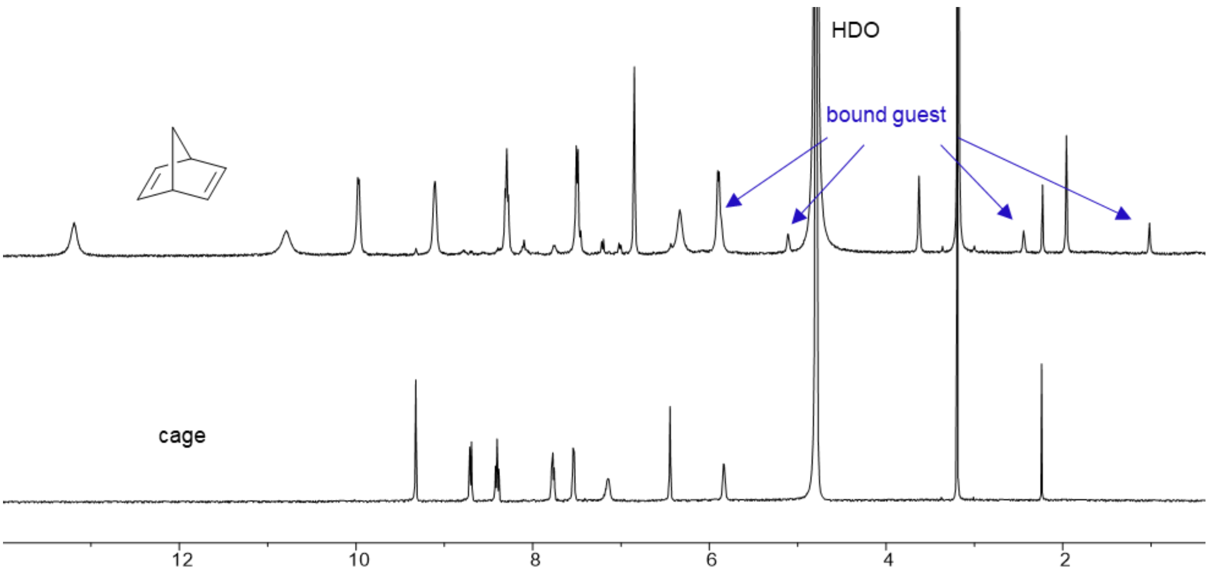

Supplementary Figure 27. ^{1}H NMR (D_2O, 400 MHz, 298 K) spectra of the $Fe^{II}{}_4L_6$ cage and norbornadiene⊂MOC.

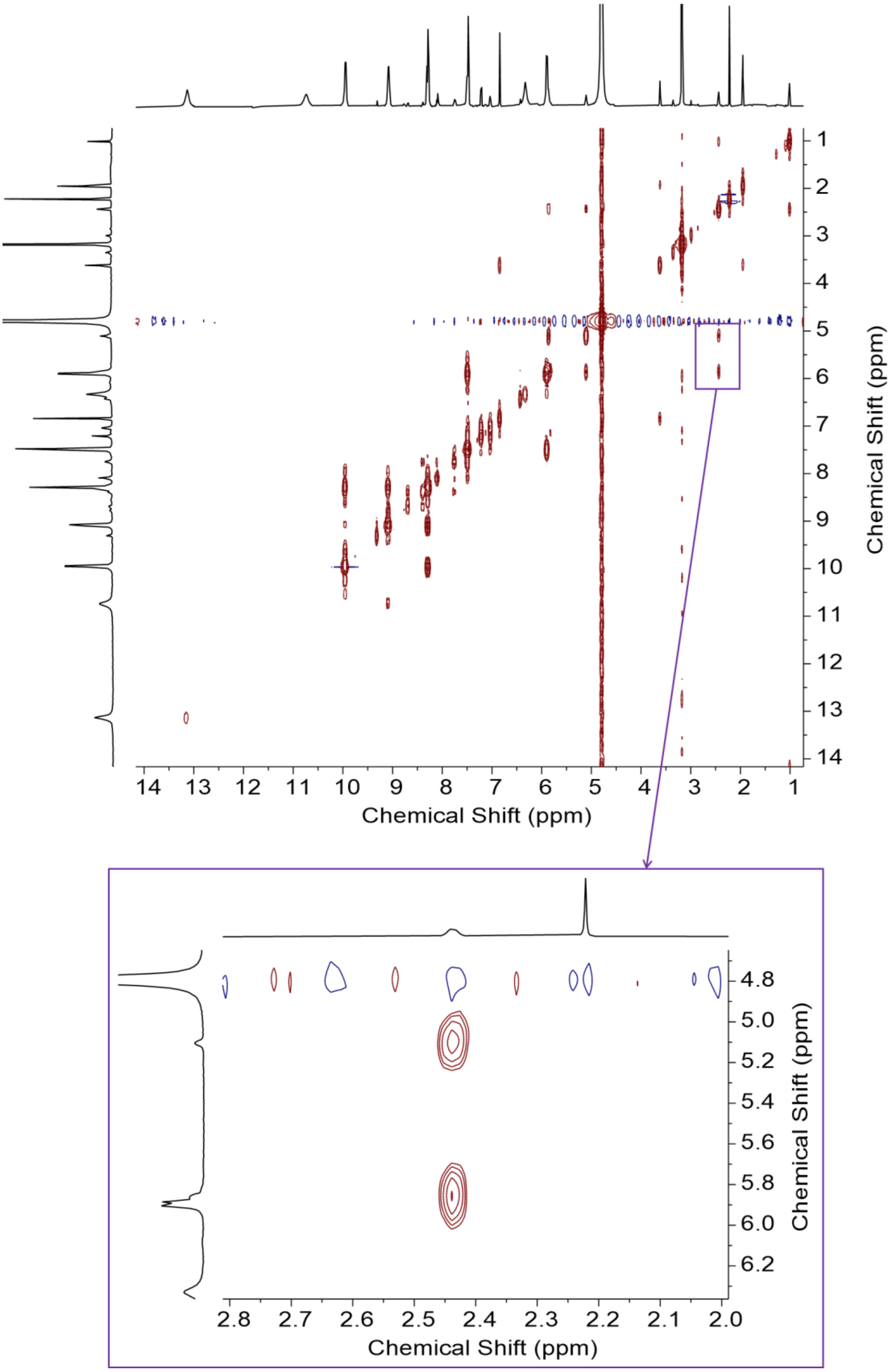

Supplementary Figure 28. ^{1}H-^{1}H COSY spectrum (D_2O, 400 MHz, 298 K) of norbornadiene⊂MOC.

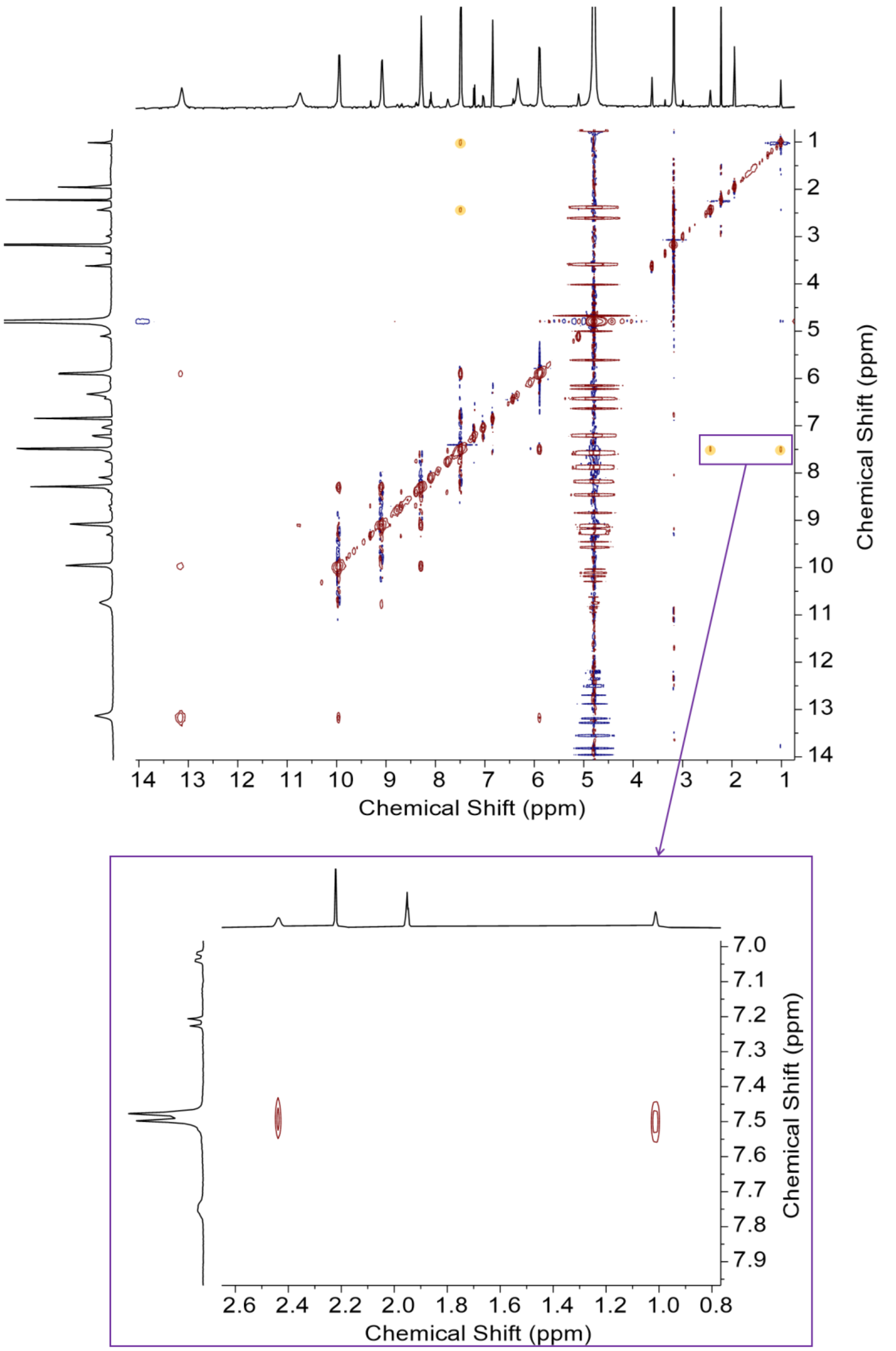

Supplementary Figure 29. ^{1}H-^{1}H NOESY spectrum (D_2O, 400 MHz, 298 K) of norbornadiene⊂MOC. The NOE peaks between the encapsulated guest protons and occupied cage have been highlighted.

3.3 Binding of norbornene

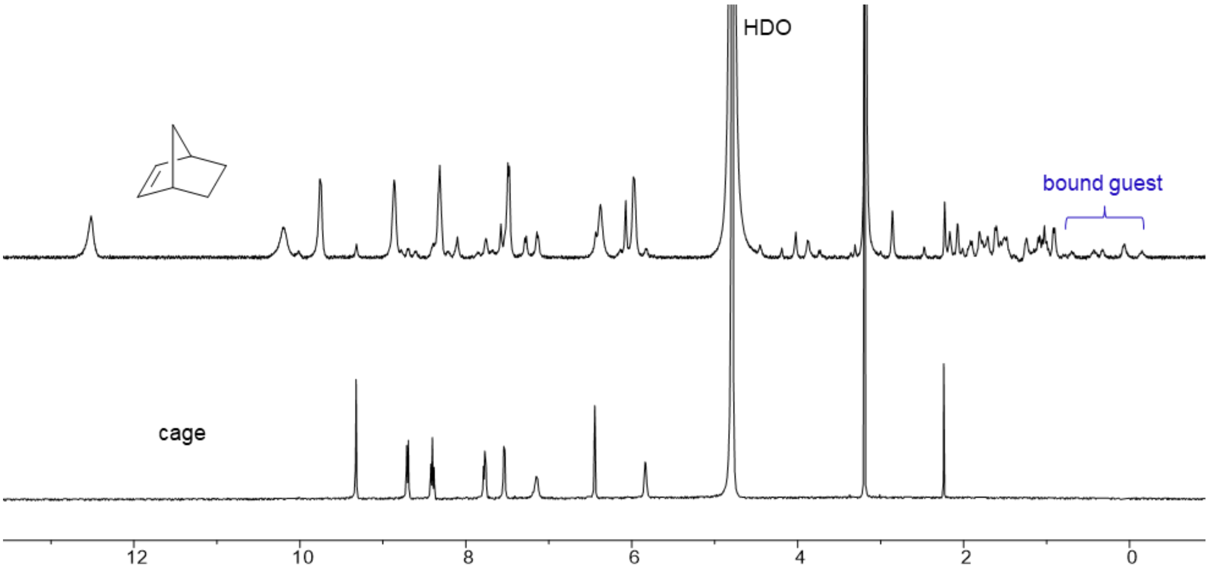

Supplementary Figure 30. ^{1}H NMR (D_2O, 400 MHz, 298 K) spectra of the $Fe^{II}{}_4L_6$ cage and norbornene⊂MOC.

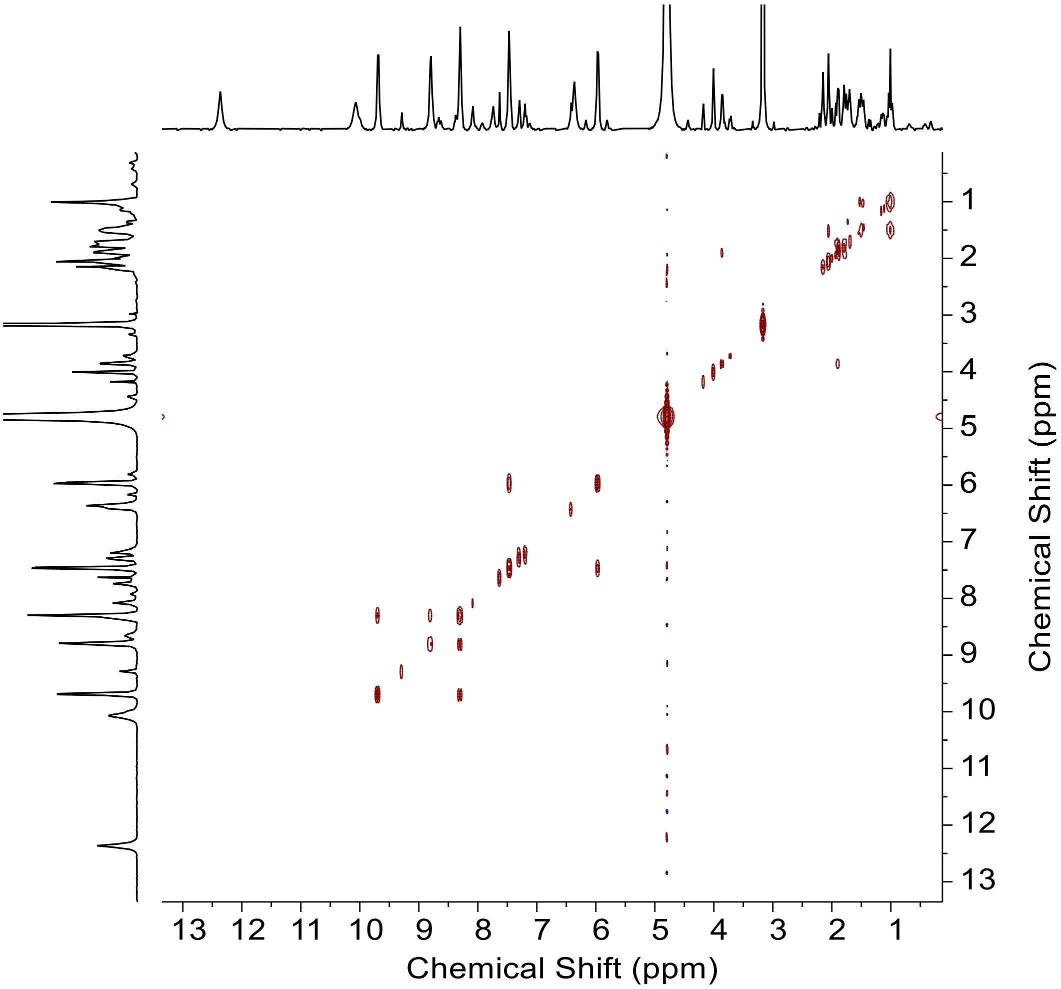

Supplementary Figure 31. ^{1}H-^{1}H COSY spectrum (D_2O, 400 MHz, 298 K) of norbornene⊂MOC.

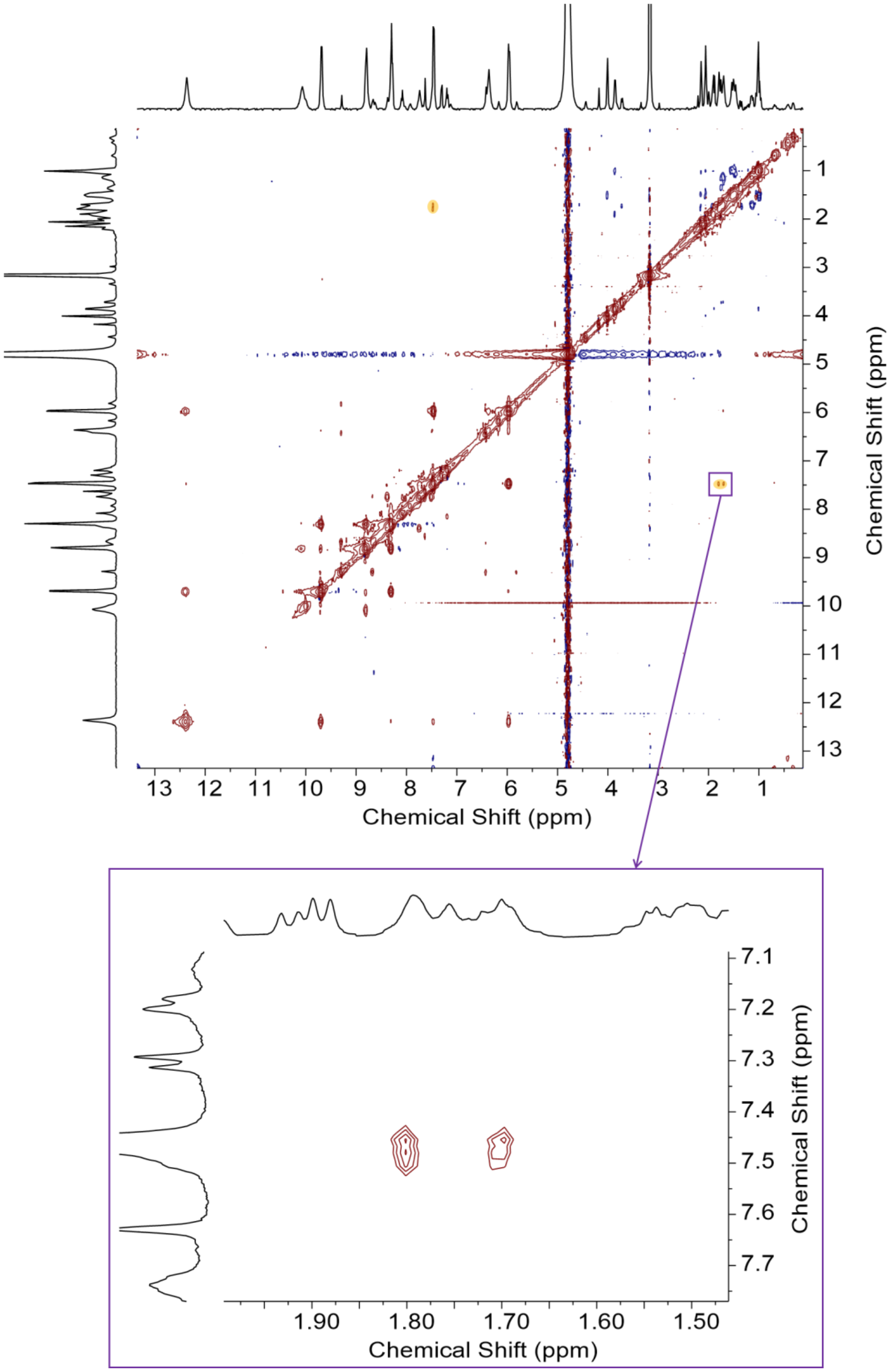

Supplementary Figure 32. ^{1}H-^{1}H NOESY spectrum (D_2O, 400 MHz, 298 K) of norbornene⊂MOC. The NOE peaks between the encapsulated guest protons and occupied cage have been highlighted.

3.4 Binding of norbornane

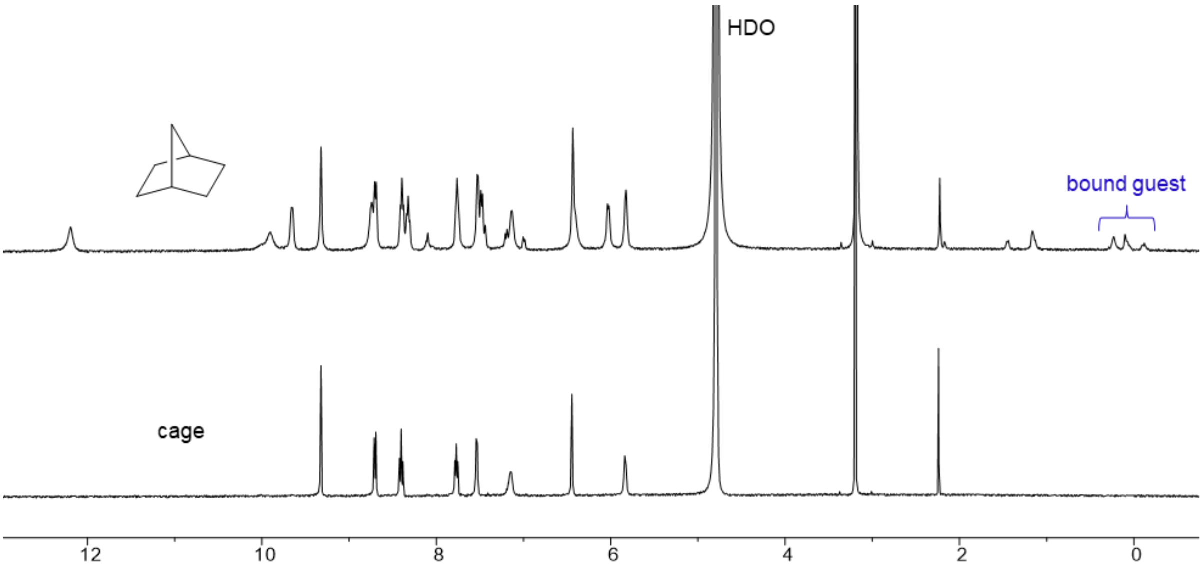

Supplementary Figure 33. ^{1}H NMR (D_2O, 400 MHz, 298 K) spectra of the $Fe^{II}{}_4L_6$ cage and norbornane⊂MOC.

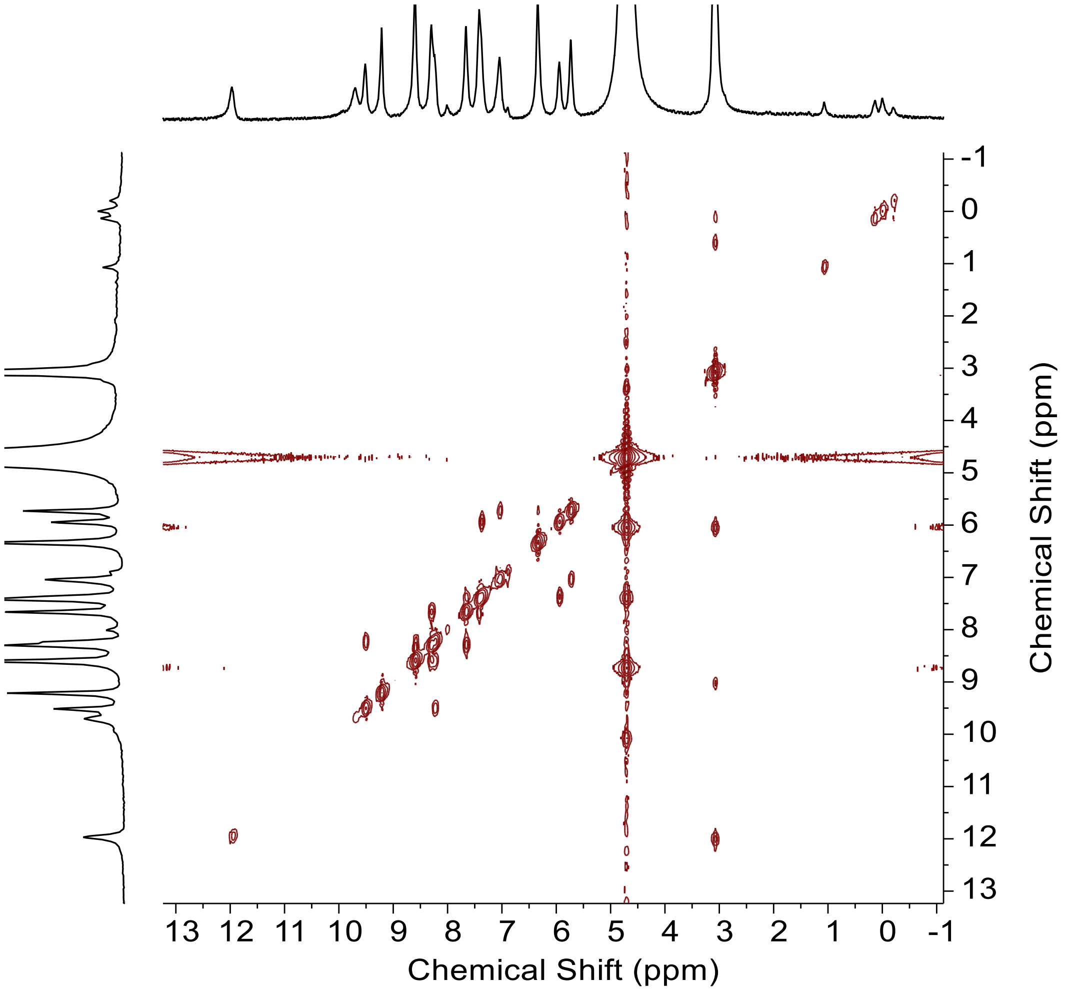

Supplementary Figure 34. ^{1}H-^{1}H COSY spectrum (D_2O, 400 MHz, 298 K) of norbornane⊂MOC.

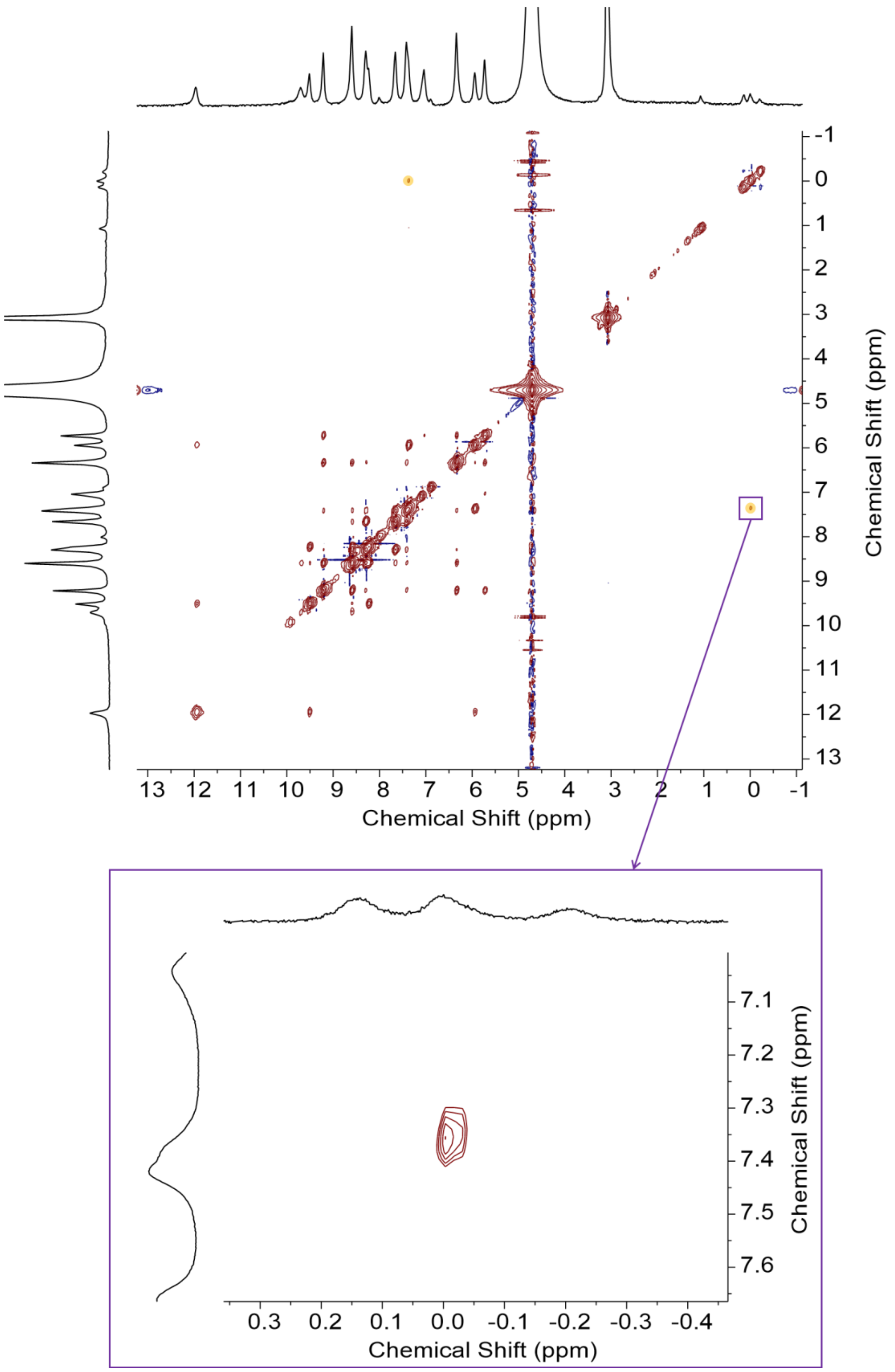

Supplementary Figure 35. ^1H-^1H NOESY spectrum (D_2O, 400 MHz, 298 K) of norbornane⊂MOC. The NOE peaks between the encapsulated guest protons and occupied cage have been highlighted.

3.5 Binding of 7-oxabicycloheptane

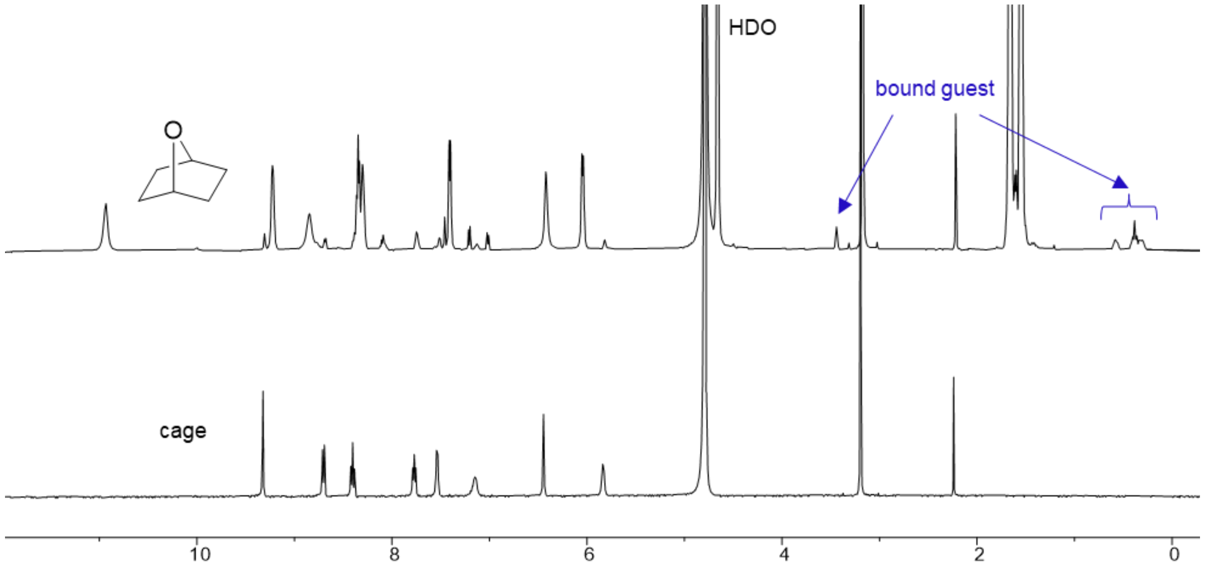

Supplementary Figure 36. ^{1}H NMR (D_2O, 400 MHz, 298 K) spectra of the $Fe^{II}{}_4L_6$ cage and 7-oxabicycloheptane⊂MOC.

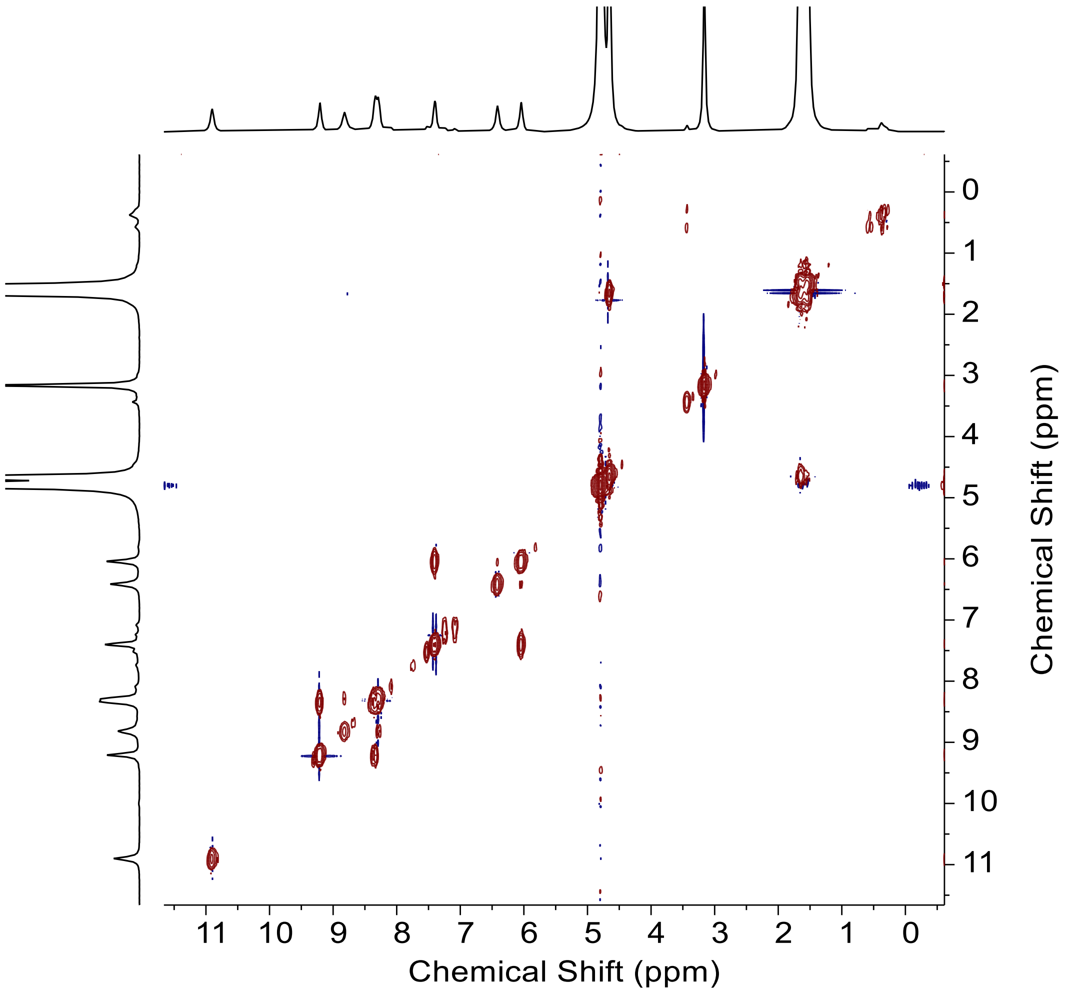

Supplementary Figure 37. ^{1}H-^{1}H COSY (D_2O, 400 MHz, 298 K) spectrum of 7-oxabicycloheptane⊂MOC.

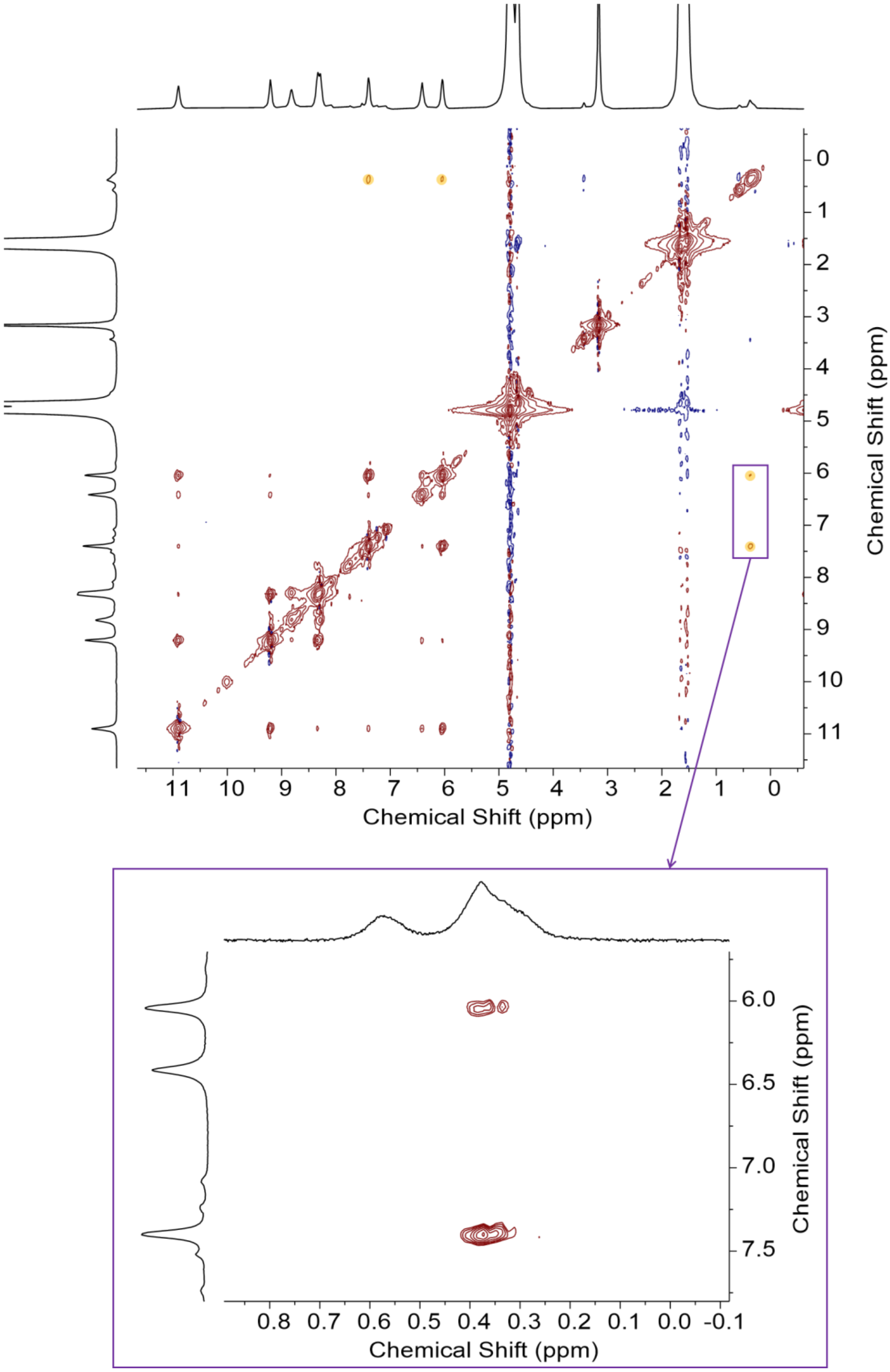

Supplementary Figure 38. ^1H-^1H NOESY (D_2O, 400 MHz, 298 K) spectrum of 7-oxabicycloheptane⊂MOC. The NOE peaks between the encapsulated guest protons and occupied cage have been highlighted.

3.6 Determination of binding constants

The binding constants for the guests listed in Supplementary Figure 24 with the $Fe^{II}_4L_6$ cage were determined following a previously described procedure.[5]

Addition of a guest into a solution of the cage established the following equilibrium:

$$H + G \rightleftarrows HG$$

The concentration of free cage **[H]**, free guest **[G]**, and the host-guest complex **[HG]** could be determined from integration of their NMR peaks relative to those of the *tert*-butanol internal standard.

The binding constant of the cage towards $CHCl_3$, benzene, norbornadiene, norbornene, norbornane, and 7-oxabicycloheptane could thus be calculated through the following equation:

$$K_a = \frac{[HG]}{[H][G]} \tag{2}$$

For 1,4-cyclohexadiene, cyclohexene and cyclohexane which bind strongly to the cage, no empty host was observed by NMR spectroscopy. To circumvent this practical problem, we prepared a solution saturated with both benzene (G_1) and the second guest of interest (G_2) in the presence of the cage. Under these conditions, there is the following equilibrium:

$$HG_1 + G_2 \rightleftarrows HG_2 + G_1$$

The relative binding constant (K_{rel}) is given by:

$$K_{rel} = \frac{[HG_2][G_1]}{[HG_1][G_2]} = \frac{[HG_2]}{[HG_1]} \times \frac{S_{max,1}}{S_{max,2}} = \frac{K_{a,2}}{K_{a,1}} \tag{3}$$

The concentration of the two host−guest complexes **[HG_1]** and **[HG_2]** could be determined from integration of their NMR peaks relative to those of the *tert*-butanol internal standard. The saturated concentrations of benzene **[$S_{max,1}$]** and the second guest **[$S_{max,2}$]** could be known from Abraham and Le.[6] The binding constant for the second guest, $K_{a,2}$, could be calculated from K_{rel} and $K_{a,1}$, since the binding constant of benzene, $K_{a,1}$, is known.

Different amounts of guests were added, and an average value of the binding constant was calculated. The binding constants of the cage towards these guests are tabulated in Supplementary Table 2.

Supplementary Table 2. Binding constants (K_a) of the $Fe^{II}_4L_6$ cage for various guests in D_2O.

guest	K_a (M^{-1}) [a]
$CHCl_3$	$(2.43\pm0.2)\times10^3$
benzene	$(2.89\pm0.1)\times10^3$
1,4-cyclohexadiene	$(2.22\pm0.2)\times10^4$
cyclohexene	$(1.43\pm0.2)\times10^4$
cyclohexane	$(4.77\pm0.1)\times10^4$
norbornadiene	$(1.25\pm0.2)\times10^4$
norbornene	$(6.22\pm0.5)\times10^3$
norbornane	$(2.29\pm0.3)\times10^3$
7-oxabicycloheptane	$(2.71\pm0.3)\times10^2$

[a] Athough the binding constants for some of the guests, including $CHCl_3$, benzene, cyclohexene, and cyclohexane, were previously reported,[5] for consistency, the binding constants for all of the guests were remeasured in this work.

3.7 Binding of norbornadiene and 7-oxabicycloheptane with the solid-state cage

Among the four new bicyclic guests, norbornadiene and 7-oxabicycloheptane are liquids. Binding experiments of the $Fe^{II}_4L_6$ cage in the solid state by adding cage solids directly into the liquids of pure guest were performed. After stirring the heterogeneous mixtures of the solid-state cages with norbornadiene or 7-oxabicycloheptane at 50 °C overnight, centrifugation was conducted to remove the liquids. The obtained solids were dissolved in D_2O and ^1H NMR spectra were measured immediately. As the binding of norbornadiene and 7-oxabicycloheptane with the dissolved cage in D_2O is slow, the immediate measurement of ^1H NMR could eliminate the possibility of binding the guest molecules initially attached on the solid surface in solution. As shown in Supplementary Figure 39, the ^1H NMR spectra of cages isolated from norbornadiene or 7-oxabicycloheptane only present signals of empty cages together with signals of free guests. We infer the poor binding ability of the solid-state cage resulted from the nonporous nature of the cage solid as well as the poor flexibility and dynamicity of the solid-state cage.

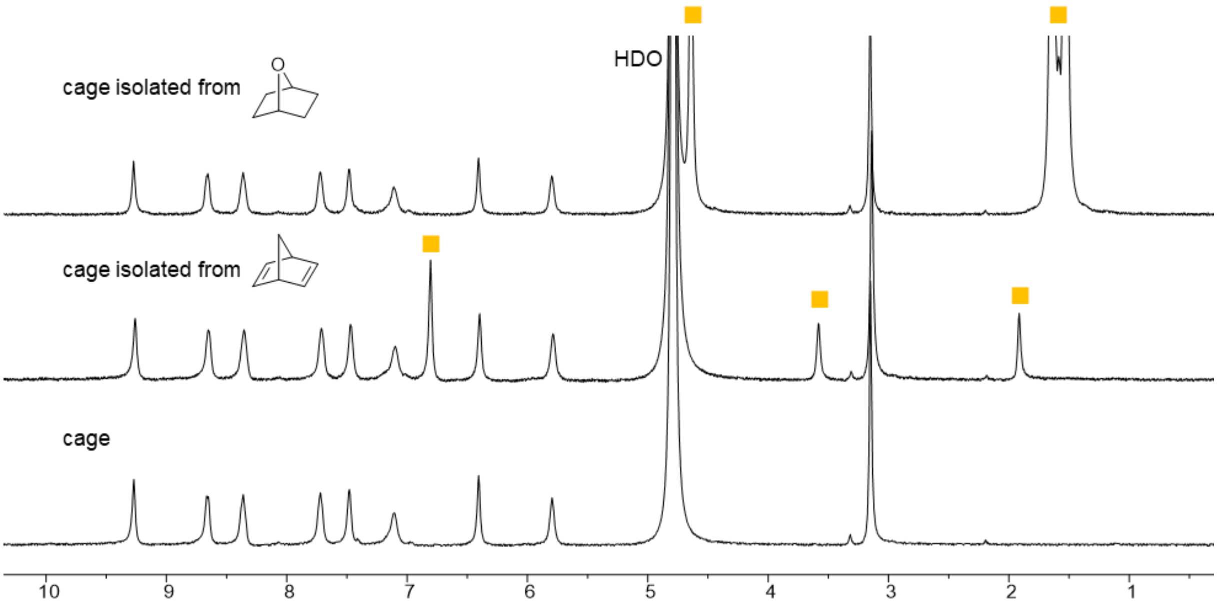

Supplementary Figure 39. ^1H NMR (D_2O, 400 MHz, 298 K) spectra of the $Fe^{II}_4L_6$ cage and the $Fe^{II}_4L_6$ cage isolated from the liquid of norbornadiene or 7-oxabicycloheptane. Peaks of free guests have been labelled with orange square.

4. Host-guest chemistry of the immobilized cage within MOC@PILs

4.1 ^{1}H NMR spectral characterization

All of the guests listed in Supplementary Figure 24 were tested to bind with the immobilized cage within MOC@PILs. Apart from fluorobenzene, the binding of which could be characterized by ^{19}F NMR directly, the binding of other guests was investigated by an ion exchange strategy (Supplementary Figure 40). After throughly mixing MOC@PILs, for instance **6**, with a guest in water and equilibrating for a few hours, centrifugation was conducted to remove the supernatant. The obtained hydrogel (guest⊂MOC)@PIL **6** was washed with water three times to remove the surface-attached molecules of free guest. An excess of $NaNO_3$ was then added into the D_2O solution of (guest⊂MOC)@PIL **6**, resulting in the release of the anionic guest⊂MOC species into solution pairing with Na^+. The supernatant containing Na_4[guest⊂MOC] was obtained through centrifugation and the ^{1}H NMR spectrum was recorded immediately.

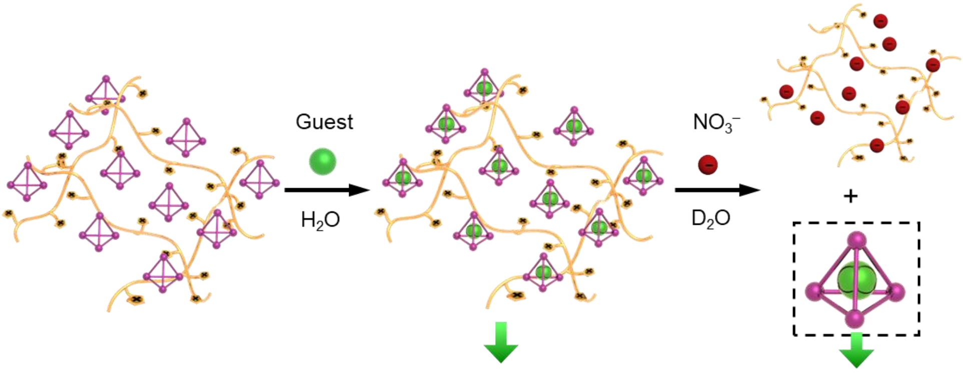

Supplementary Figure 40. Schematic illustration of the strategy for investigation of the host-guest chemistry of MOC@PILs.

Binding of CH_2Cl_2

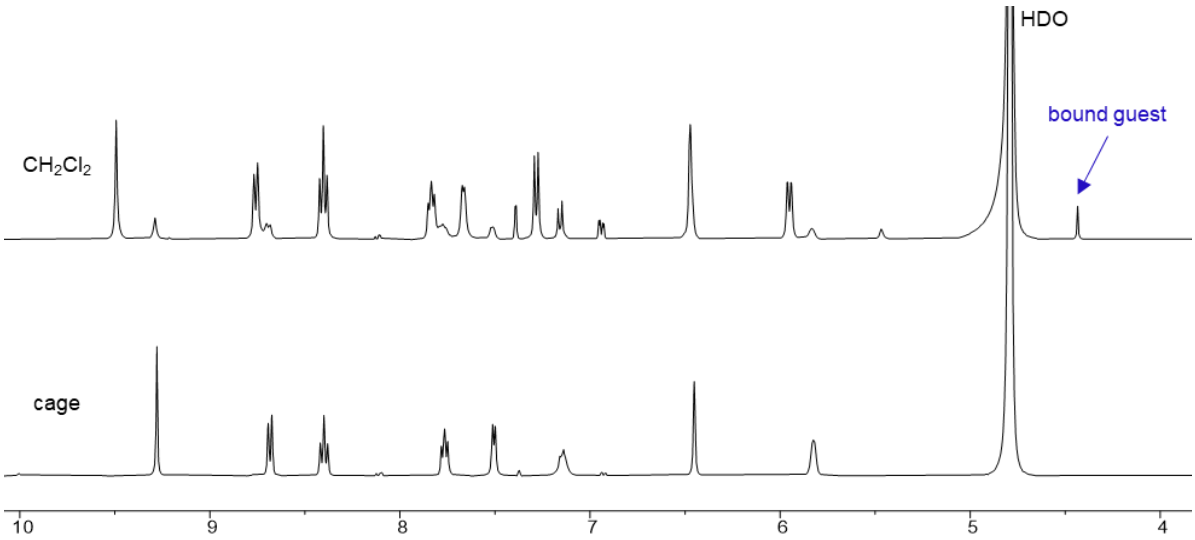

Supplementary Figure 41. ^{1}H NMR (D_2O, 400 MHz, 298 K) spectra of the anionic cage and CH_2Cl_2⊂MOC released from MOC@PIL **6** or (guest⊂MOC)@PIL **6** by adding excess $NaNO_3$.

Binding of $CHCl_3$

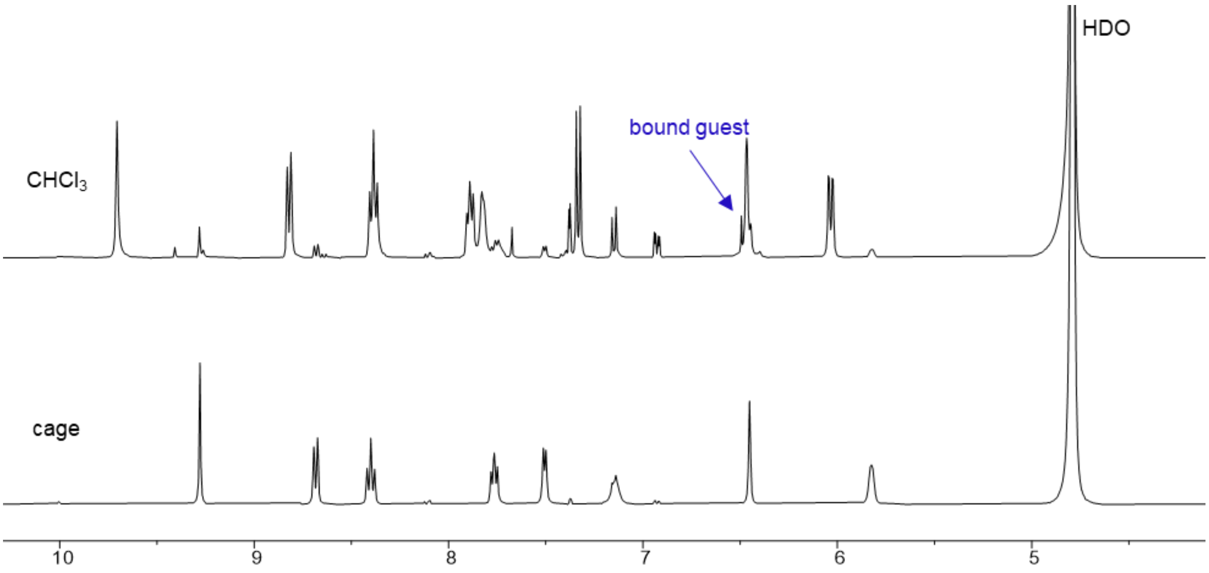

Supplementary Figure 42. ^1H NMR (D_2O, 400 MHz, 298 K) spectra of the anionic cage and $CHCl_3$⊂MOC released from MOC@PIL **6** or (guest⊂MOC)@PIL **6** by adding excess $NaNO_3$.

Binding of 1,4-dioxane

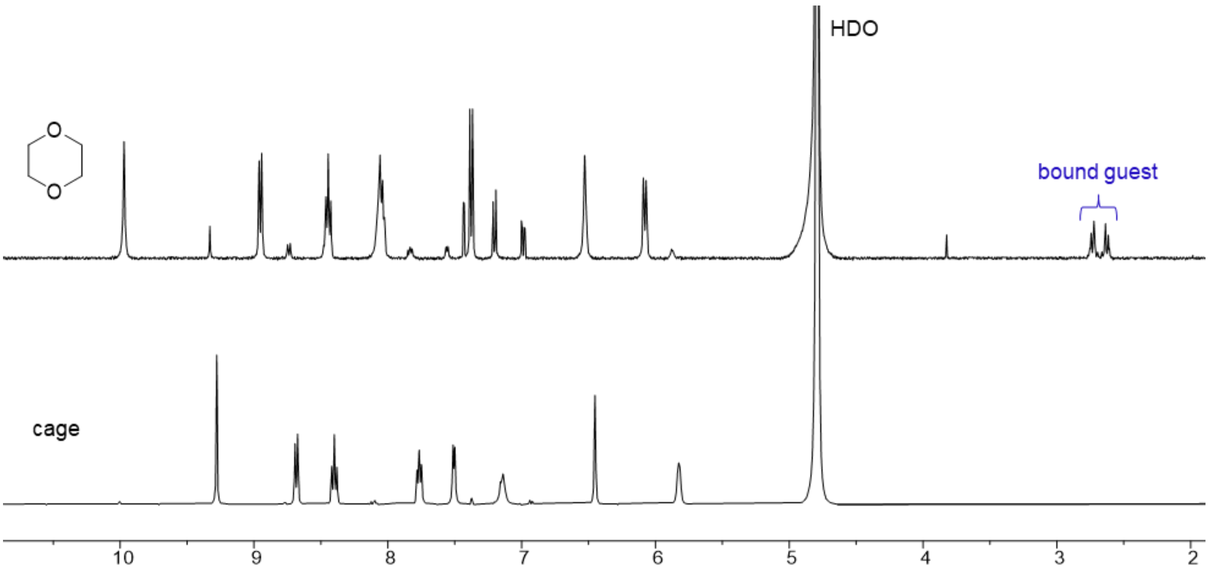

Supplementary Figure 43. ^1H NMR (D_2O, 400 MHz, 298 K) spectra of the anionic cage and 1,4-dioxane⊂MOC released from MOC@PIL **6** or (guest⊂MOC)@PIL **6** by adding excess $NaNO_3$.

Binding of benzene

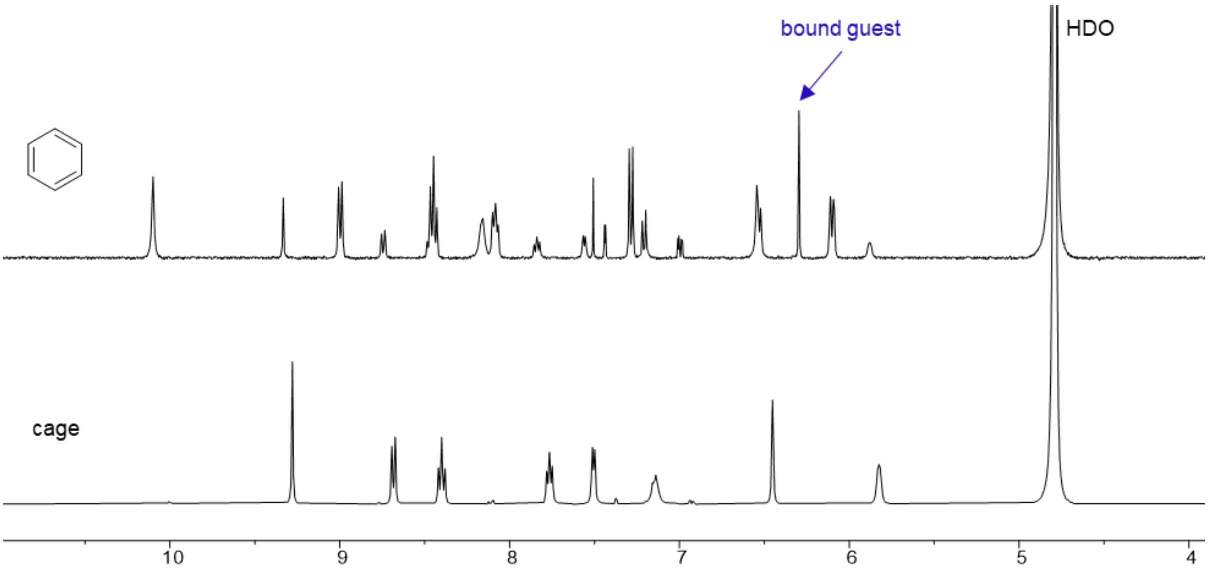

Supplementary Figure 44. ^{1}H NMR (D_2O, 400 MHz, 298 K) spectra of the anionic cage and benzene⊂MOC released from MOC@PIL **6** or (guest⊂MOC)@PIL **6** by adding excess $NaNO_3$.

Binding of 1,4-cyclohexadiene

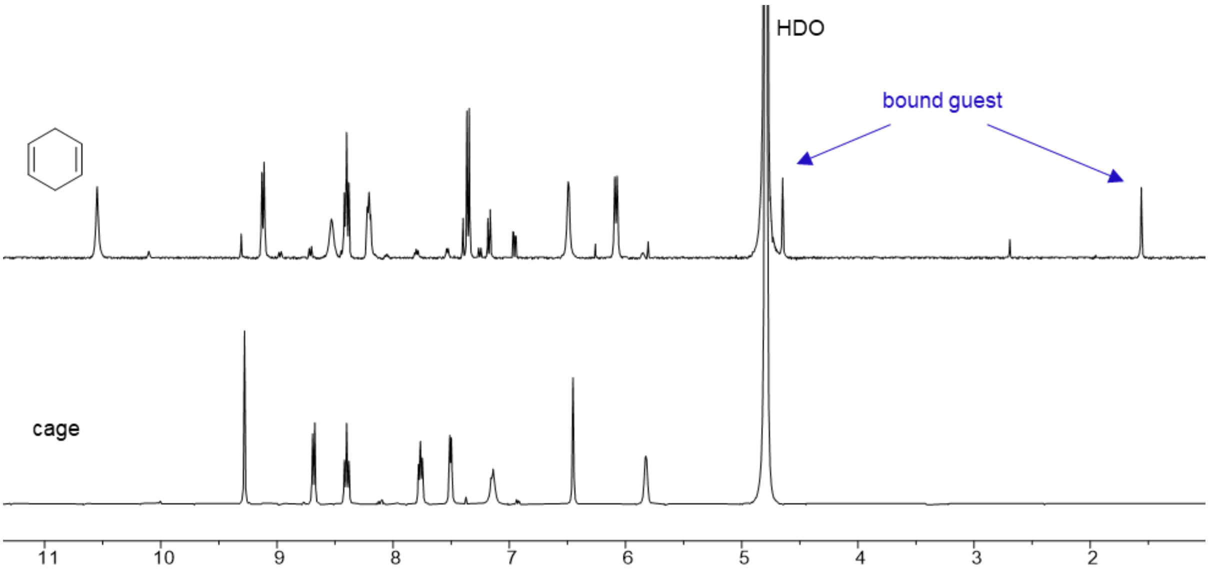

Supplementary Figure 45. ^{1}H NMR (D_2O, 400 MHz, 298 K) spectra of the anionic cage and 1,4-cyclohexadiene⊂MOC released from MOC@PIL **6** or (guest⊂MOC)@PIL **6** by adding excess $NaNO_3$.

Binding of cyclohexene

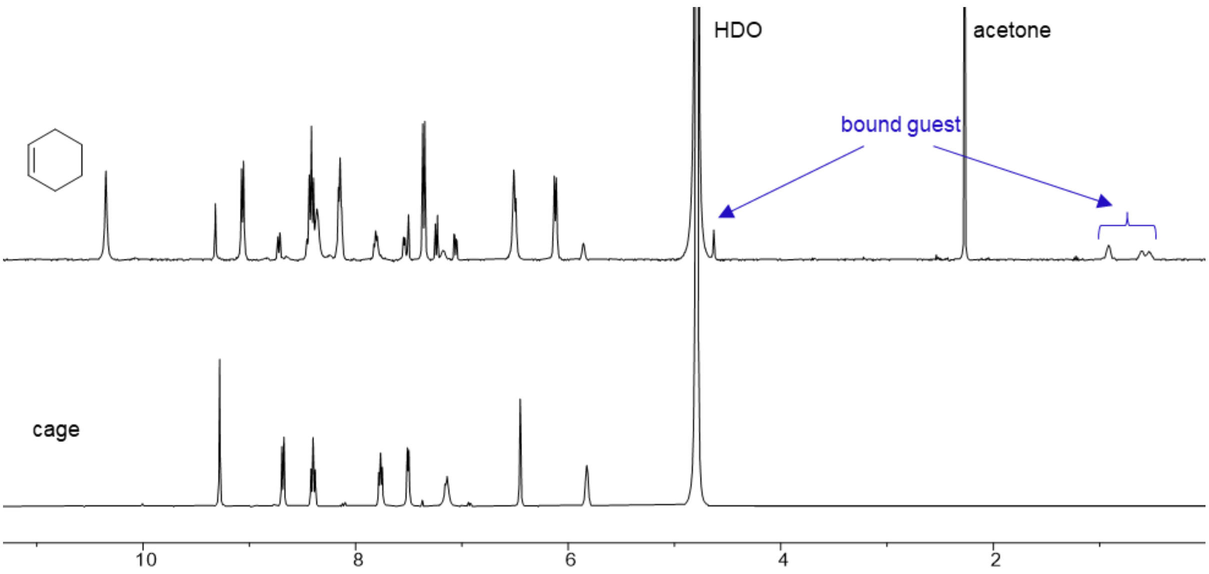

Supplementary Figure 46. ^{1}H NMR (D_2O, 400 MHz, 298 K) spectra of the anionic cage and cyclohexene⊂MOC released from MOC@PIL **6** or (guest⊂MOC)@PIL **6** by adding excess $NaNO_3$.

Binding of cyclohexane

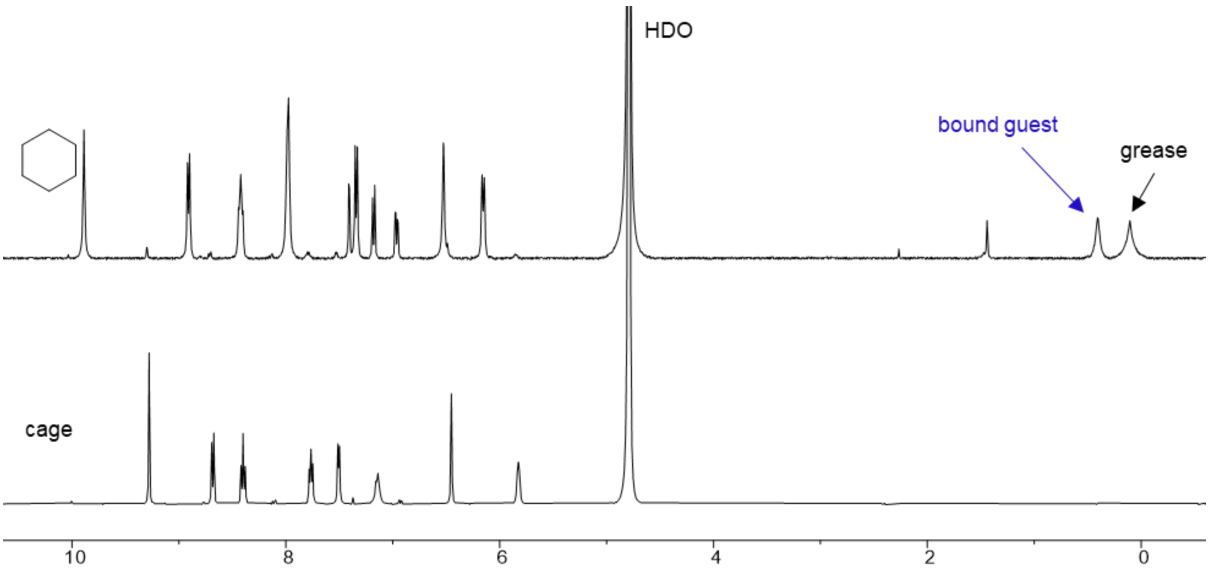

Supplementary Figure 47. ^{1}H NMR (D_2O, 400 MHz, 298 K) spectra of the anionic cage and cyclohexane⊂MOC released from MOC@PIL **6** or (guest⊂MOC)@PIL **6** by adding excess $NaNO_3$.

Binding of norbornadiene

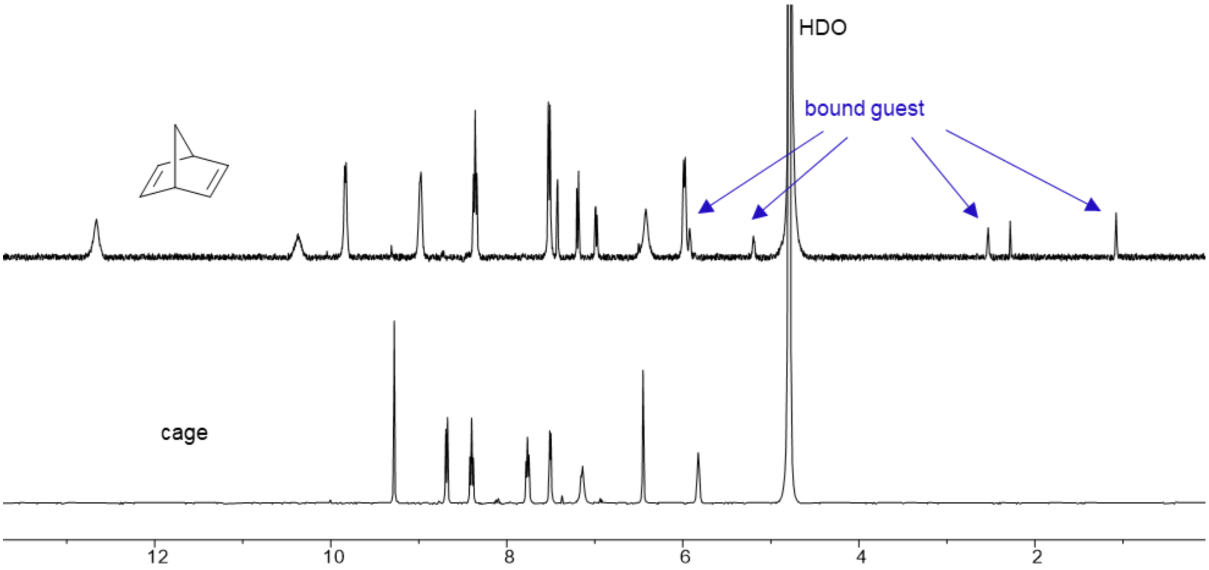

Supplementary Figure 48. ^{1}H NMR (D_2O, 400 MHz, 298 K) spectra of the anionic cage and norbornadiene⊂MOC released from MOC@PIL **6** or (guest⊂MOC)@PIL **6** by adding excess $NaNO_3$.

Binding of norbornene

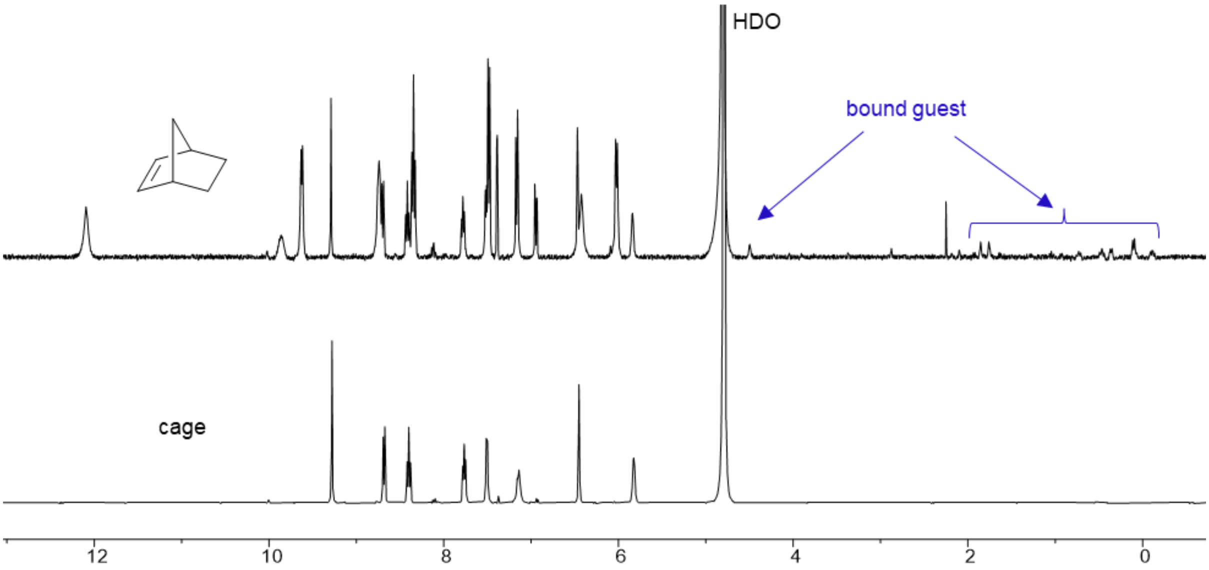

Supplementary Figure 49. ^{1}H NMR (D_2O, 400 MHz, 298 K) spectra of the anionic cage and norbornene⊂MOC released from MOC@PIL **6** or (guest⊂MOC)@PIL **6** by adding excess $NaNO_3$.

Binding of norbornane

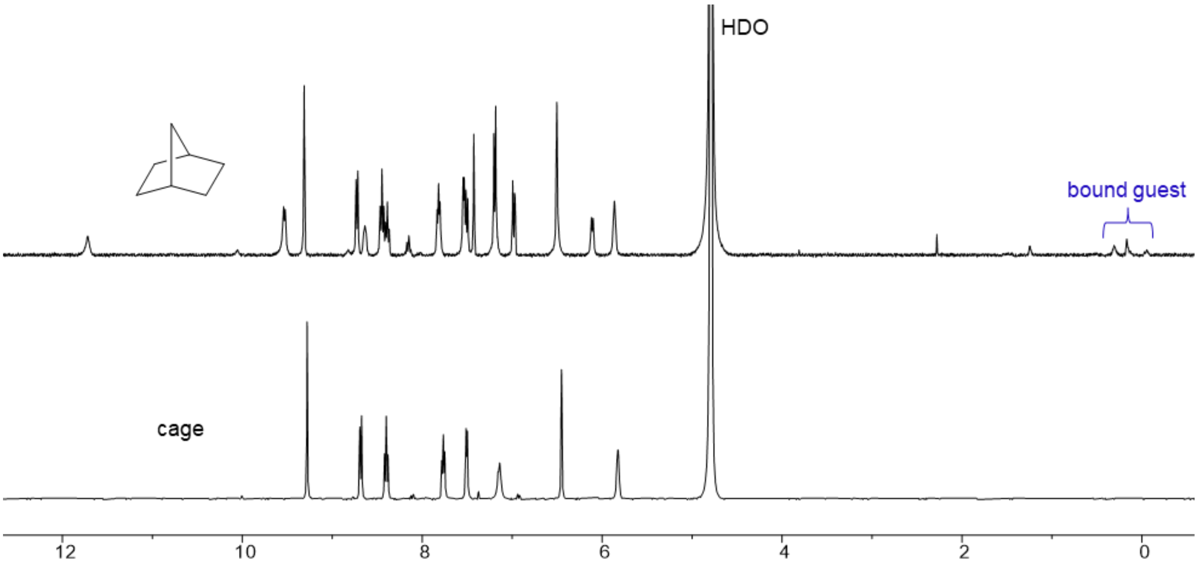

Supplementary Figure 50. ^{1}H NMR (D_2O, 400 MHz, 298 K) spectra of the anionic cage and norbornane⊂MOC released from MOC@PIL **6** or (guest⊂MOC)@PIL **6** by adding excess $NaNO_3$.

Binding of 7-oxabicycloheptane

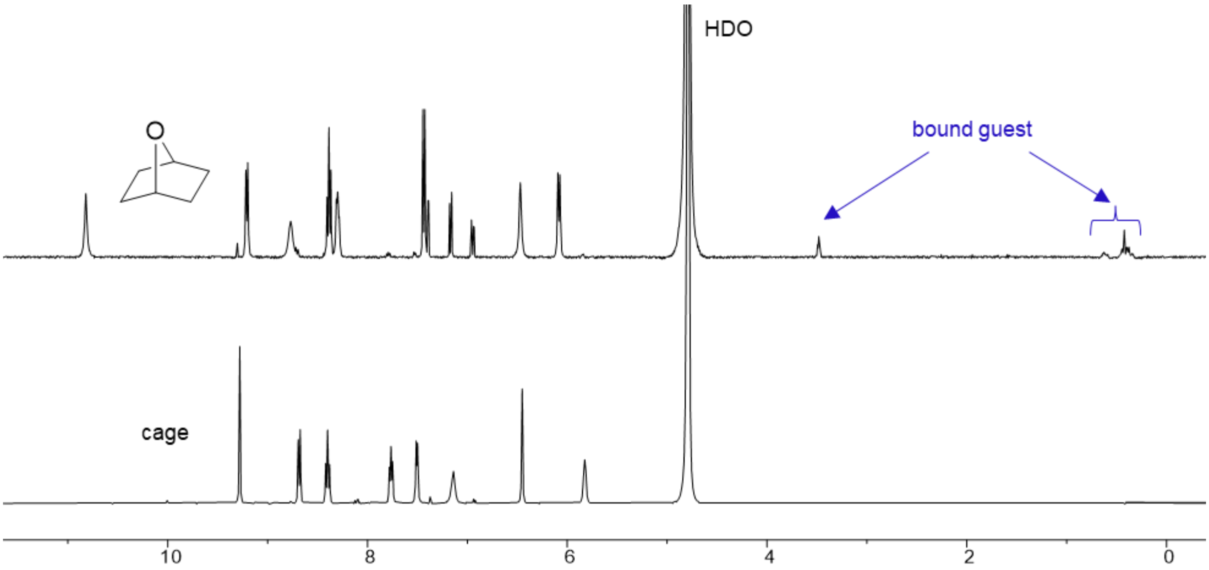

Supplementary Figure 51. ^{1}H NMR (D_2O, 400 MHz, 298 K) spectra of the anionic cage and 7-oxabicycloheptane⊂MOC released from MOC@PIL **6** or (guest⊂MOC)@PIL **6** by adding excess $NaNO_3$.

4.2 Binding kinetics

To evaluate the influence of swelling of MOC@PILs on guest binding kinetics, we monitored the ratio of $[HG]/[H]_0$ as a function of time, where [HG] and $[H]_0$ respectively represent the concentrations of the host-guest complex and the initial free host (*i.e.* total host), through ^1H NMR measurement. For the measurement of the binding kinetic curves of the soluble cage, a concentration of 1 mM cage in a 0.5 mL guest-saturated D_2O solution was used and the ^1H NMR spectra were measured after periods of time. For the measurement of the binding kinetic curves of the immobilized cage, the same amount of cage within MOC@PIL **6** as for the soluble cage was calculated and used. Similarly, MOC@PIL **6** was added into a 0.5 mL guest-saturated D_2O solution. After stirring for a period of time, the solid was separated through centrifugation and was washed with water three times to remove the surface-attached molecules of free guest. The release of cage species from MOC@PIL **6** into solution, including both guest⊂MOC and empty cage, was achieved by adding an excess of $NaNO_3$ and the corresponding ^1H NMR spectrum was recorded. Three guests, benzene, cyclohexane, and norbornane, were respectively investigated following the methods described above. Note that the binding of benzene with whichever the soluble or the immobilized cage was treated at rt, while the binding of the other two larger guests were conducted at 50 °C to facilitate the binding equilibration.

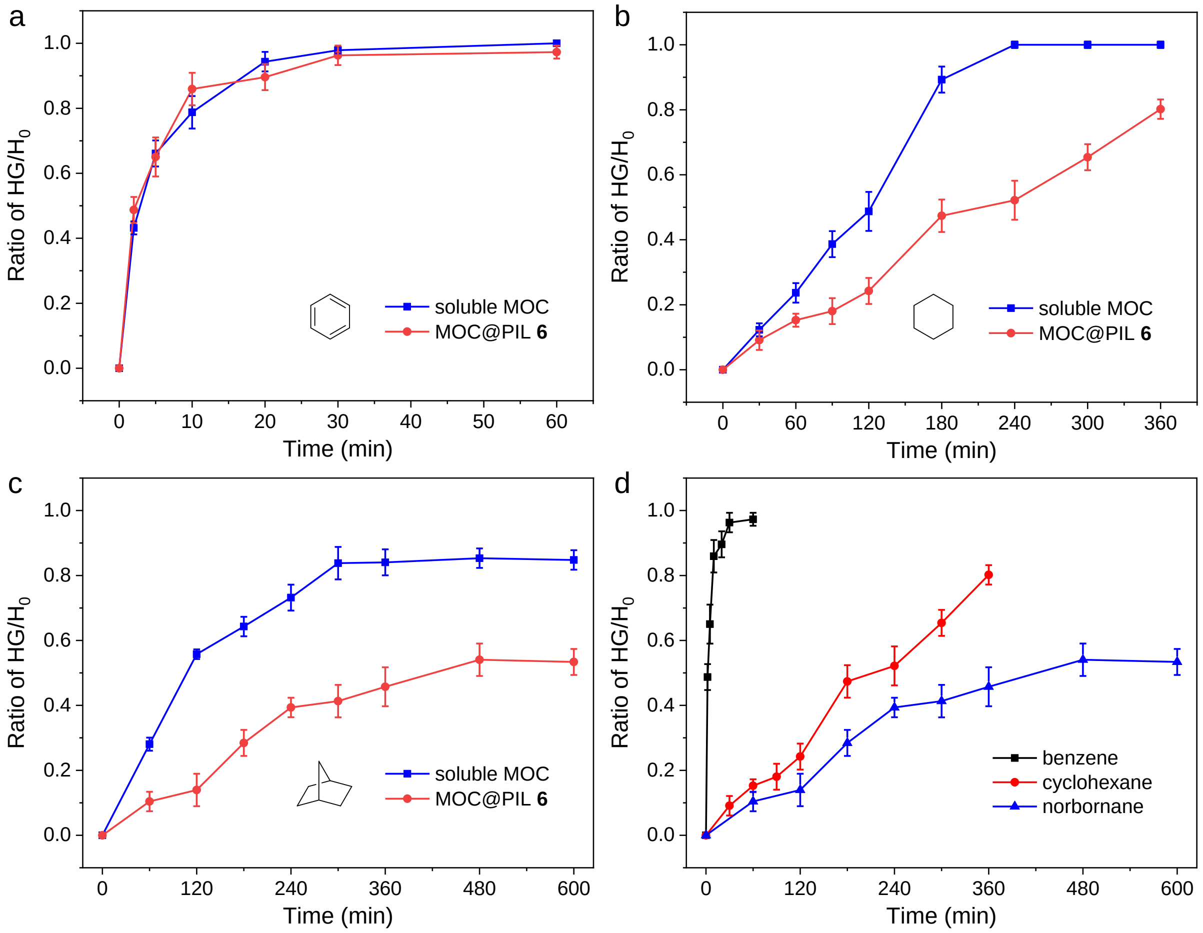

Supplementary Figure 52. Binding kinetics of the soluble cage and swollen MOC@PIL **6** for benzene (a), cyclohexane (b), and norbornane (c). Binding kinetics of the swollen MOC@PIL **6** for benzene, cyclohexane, and norbornane (d). Error ranges were calculated from triplicate experiments.

We also monitored the concentration decrease of the free guests in the presence of swollen MOC@PILs to reveal the guest uptake kinetics. For benzene, cyclohexane, and norbornane, a concentration of 10 ppm in 20 mL water was prepared individually, and MOC@PIL **2**, **4**, or **6** was added into the aqueous solution for guest uptake (the molar ratio between immobilized cage and guest was 1.5 in each case). After stirring for periods of time, GC instrument equipped with a DB-WAX UI column was used to monitor the concentration of the guest in the solution. Note that the uptake of benzene with the immobilized cage was treated at rt, while the uptake of the other two larger guests were conducted at 50 °C to facilitate the binding equilibration.

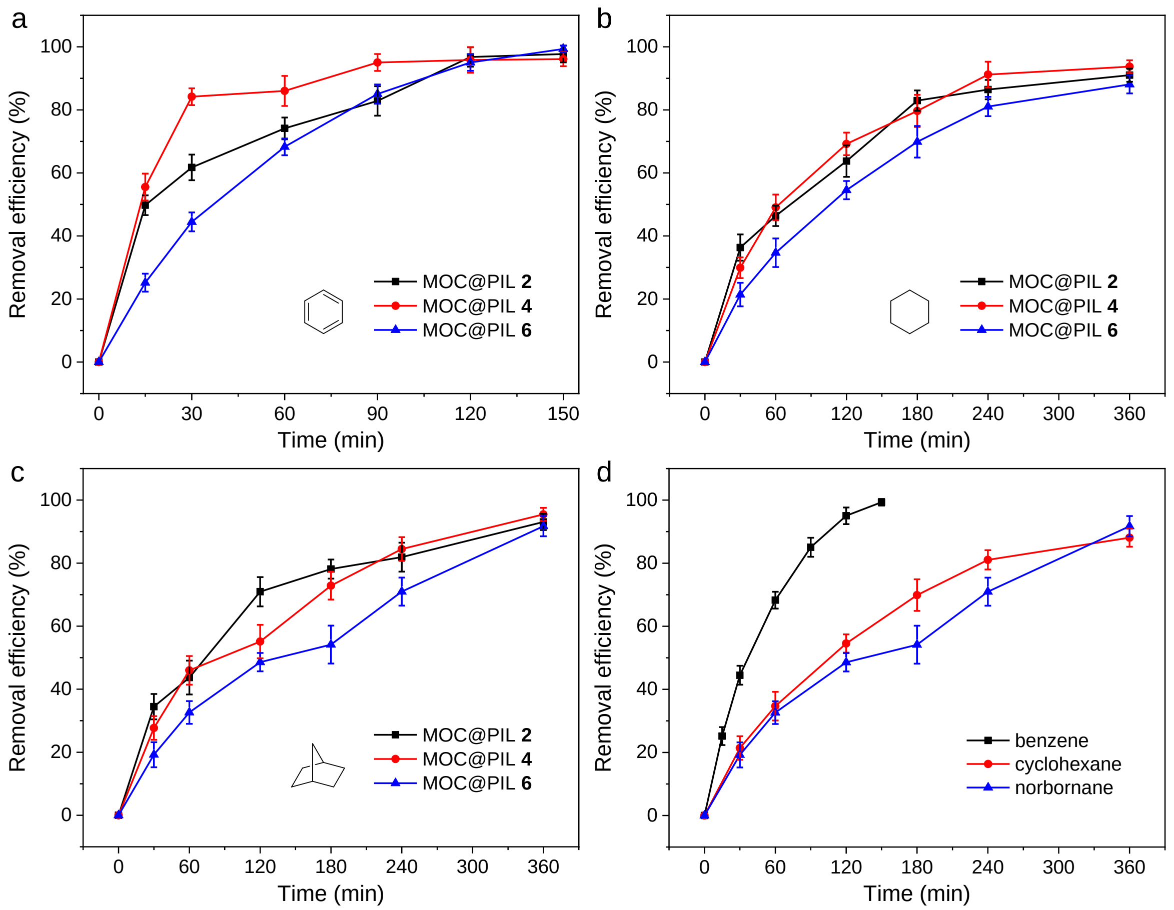

Supplementary Figure 53. Removal efficiencies of MOC@PILs **2**, **4**, **6** for benzene (a), cyclohexane (b), and norbornane (c). Removal efficiencies of MOC@PIL **6** for benzene, cyclohexane, and norbornane (d). Error ranges were calculated from triplicate experiments.

4.3 Binding thermodynamics

Apparent binding constants of the immobilized cage within MOC@PILs for the guests listed in Supplementary Figure 24 were determined through NMR experiments. After thoroughly mixing MOC@PILs, for instance **6**, with a guest in D_2O and equilibrating for a few hours, an excess of $NaNO_3$ was added into the mixture. The anionic guest⊂MOC (**HG**) together with the anionic empty cage (**H**) was exchanged by NO_3^- into the solution. Centrifugation was conducted to obtain the supernatant and the corresponding ^1H NMR spectrum was recorded immediately.

The apparent binding constants (K_a) of MOC@PILs towards $CHCl_3$, benzene, norbornadiene, norbornane, and 7-oxabicycloheptane could be determined through the following equation:

$$K_a = \frac{[HG]}{[H][G]} \tag{2}$$

The ratio between the concentration of the host-guest complex **[HG]** and the free cage **[H]** could be known from the integration ratio of the corresponding ^1H NMR peaks. The concentration of free guest **[G]** could be determined from integration of the NMR peaks relative to those of the *tert*-butanol internal standard. Note that although the addition of NO_3^- was unable to exchange all the cage species on the polymer chains into the solution, the ratio between the released guest⊂MOC and the released empty cage in solution, *i.e.* [HG]/[H], should be the same as the initial ratio on the polymer chains. The apparent binding constants determined are thus reliable.

The binding constants of MOC@PILs towards 1,4-cyclohexadiene, cyclohexene, and cyclohexane, which have lower solubility in water, were determined through the following equation:

$$K_{rel} = \frac{[HG_2][G_1]}{[HG_1][G_2]} = \frac{[HG_2]}{[HG_1]} \times \frac{S_{max,1}}{S_{max,2}} = \frac{K_{a,2}}{K_{a,1}} \tag{3}$$

The ratio between the concentrations of the two host−guest complexes **[HG_2]** and **[HG_1]** could be known from the integration ratio of the corresponding ^1H NMR peaks. The saturated concentrations of benzene **[$S_{max,1}$]** and the second guest **[$S_{max,2}$]** could be known from Abraham and Le.[6] Similarly, although the addition of NO_3^- was unable to exchange all the cage species on the polymer chains into the solution, the ratio between the released **HG_2** and **HG_1** in solution, *i.e.* $[HG_2]/[HG_1]$, should be the same as the initial ratio on the polymer chains. The apparent binding constant for the second guest, $K_{a,2}$, could be calculated from K_{rel} and $K_{a,1}$, since the apparent binding constant of benzene, $K_{a,1}$, is known.

The apparent binding constants of the immobilized cage within MOC@PILs towards these guests are tabulated in Supplementary Tables S3 and S4.

Supplementary Table 3. Binding constants (K_a) of the soluble cage and apparent binding constants (apparent K_a) of the immobilized cage within MOC@PIL **6** for various guests in D_2O.

guest	K_a (M^{-1})	apparent K_a (M^{-1})[b]
$CHCl_3$	2.43×10^3	2.7×10^3
benzene	2.89×10^3	2.7×10^3
1,4-cyclohexadiene	2.22×10^4	1.5×10^4
cyclohexene	1.43×10^4	6.9×10^3
cyclohexane	4.77×10^4	4.1×10^3
norbornadiene	1.25×10^4	3.4×10^3
norbornene	6.22×10^3	n.d.[a]
norbornane	2.29×10^3	3.8×10^2
7-oxabicycloheptane	2.71×10^2	3.4×10^2

[a] The apparent binding constant could not be determined due to the difficulty in the assignment of peaks of the encapsulated guest on the ^1H NMR spectrum.
[b] Due to the complicated procedures of the measurement, data are presented with only two significant figures to avoid inaccuracies. All errors are below 10%.

Supplementary Table 4. Binding constants (K_a) of the free cage and apparent binding constants (apparent K_a) of the immobilized cages within MOC@PILs **1-6** for benzene and norbornane guests, respectively.

host	benzene (M^{-1})	norbornane (M^{-1})
free cage	2.89×10^3	2.29×10^3
MOC@PIL **1**[a]	2.8×10^3	1.8×10^3
MOC@PIL **2**[a]	3.6×10^3	1.6×10^3
MOC@PIL **3**[a]	3.3×10^3	1.1×10^3
MOC@PIL **4**[a]	2.7×10^3	1.0×10^3
MOC@PIL **5**[a]	2.9×10^3	0.7×10^3
MOC@PIL **6**[a]	2.7×10^3	0.4×10^3

[a] Due to the complicated procedures of the measurement, data are presented with only two significant figures to avoid inaccuracies. All errors are below 10%.

5. Pollutants removal from water

5.1 Procedures and removal efficiency

MOC@PIL **6** was selected for the removal of organic pollutants from water due to the high cage loading, appropriate swellability and stiffness for recycling. For pollutants CH_2Cl_2, $CHCl_3$, benzene, 1,4-cyclohexadiene, 1,4-dioxane, cyclohexene, cyclohexane, norbornadiene, and 7-oxabicycloheptane, a concentration of 5 mM in water was prepared individually, and dry MOC@PIL **6** (40 mg) was added into the aqueous solution (1.5 mL) for adsorption (the molar ratio between immobilized cage and pollutant was 1.2). For pollutants norbornene and norbornane, a concentration of 1.34 and 1.46 mM was respectively prepared due to their lower solubility in water, and the same amount of MOC@PIL **6** (40 mg) was used for adsorption. After complete swelling and adsorption, centrifugation was conducted and the supernatant, corresponding to the purified water, was collected. The concentration of the pollutant after adsorption was analyzed by ^{1}H NMR spectroscopy, and *tert*-butanol or ethylene glycol was used as the internal standard. The simulated polluted water containing CH_2Cl_2, $CHCl_3$, benzene, or 1,4-cyclohexadiene was treated at rt for 2 h for adsorption, while the adsorption of 1,4-dioxane, cyclohexene, cyclohexane, norbornadiene, norbornene, norbornane, or 7-oxabicycloheptane was conducted at 50 °C for 6 h to facilitate equilibration. For comparison, control experiments were carried out under the same conditions except the use of 40 mg PIL-PF_6^- as the adsorbent or without adding any adsorbents. These control experiments could demonstrate the critical role of the immobilized cage for adsorption, ruling out the effect of other factors.

Adsorption of CH_2Cl_2

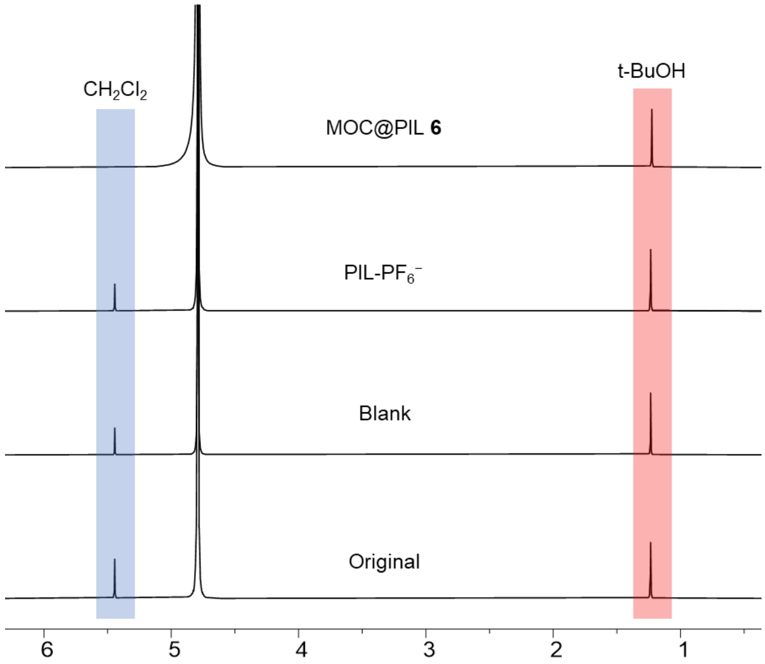

Supplementary Figure 54. ^{1}H NMR (D_2O, 400 MHz, 298 K, number of scans = 64) spectra of the polluted water before and after adsorption of CH_2Cl_2. The initial concentration of CH_2Cl_2 was 5 mM.

Adsorption of $CHCl_3$

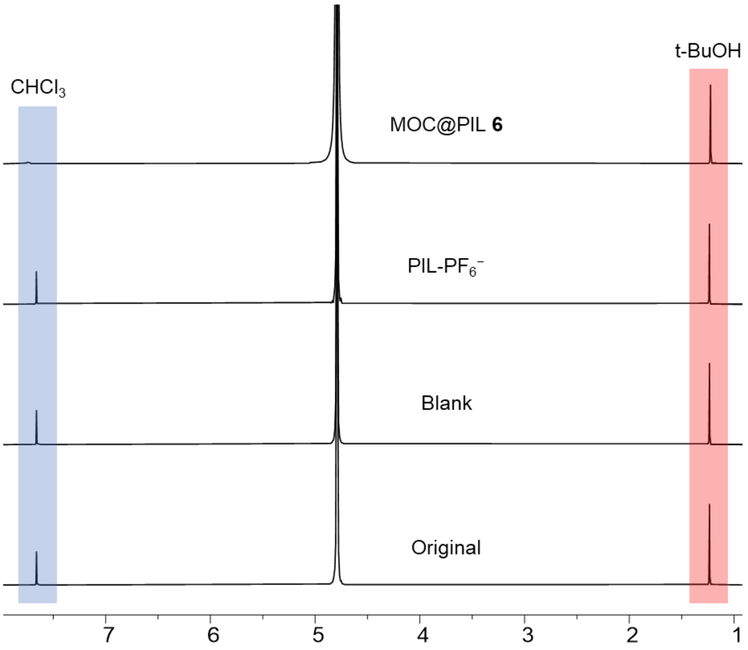

Supplementary Figure 55. ^{1}H NMR (D_2O, 400 MHz, 298 K, number of scans = 64) spectra of the polluted water before and after adsorption of $CHCl_3$. The initial concentration of $CHCl_3$ was 5 mM.

Adsorption of benzene

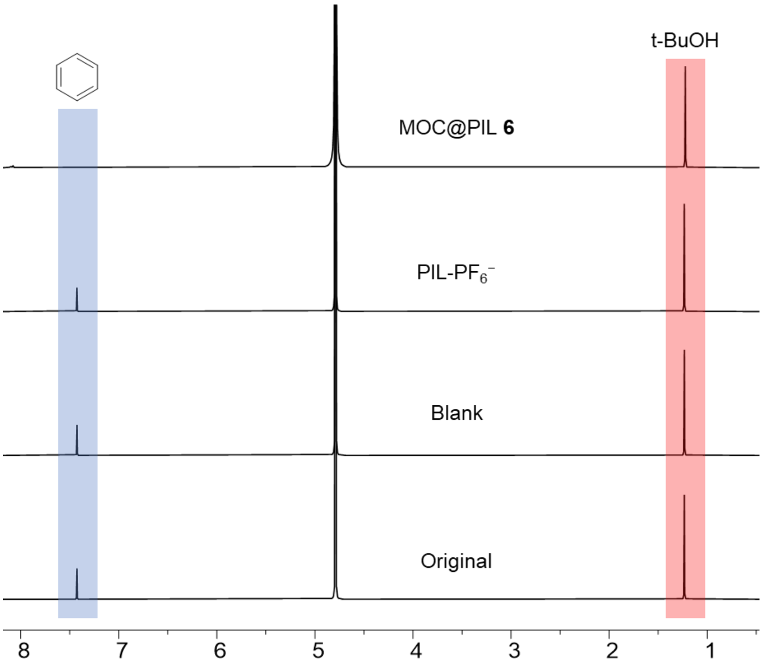

Supplementary Figure 56. ^{1}H NMR (D_2O, 400 MHz, 298 K, number of scans = 64) spectra of the polluted water before and after adsorption of benzene. The initial concentration of benzene was 5 mM.

Adsorption of 1,4-cyclohexadiene

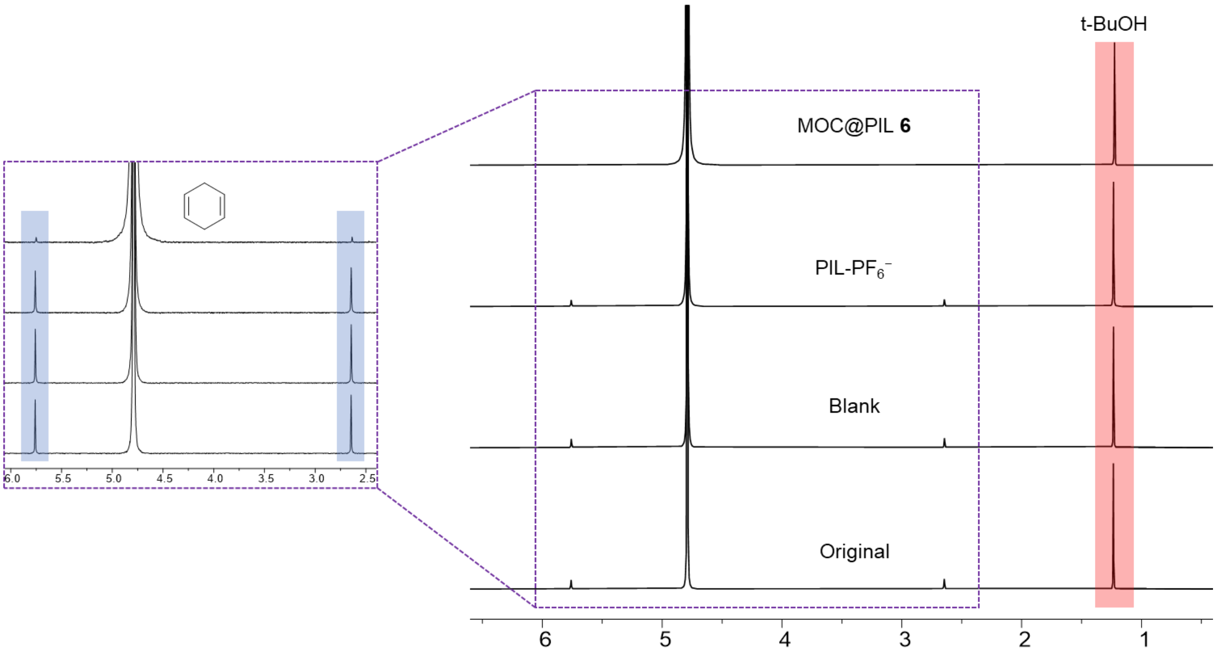

Supplementary Figure 57. ^{1}H NMR (D_2O, 400 MHz, 298 K, number of scans = 64) spectra of the polluted water before and after adsorption of 1,4-cyclohexadiene. The initial concentration of 1,4-cyclohexadiene was 5 mM.

Adsorption of 1,4-dioxane

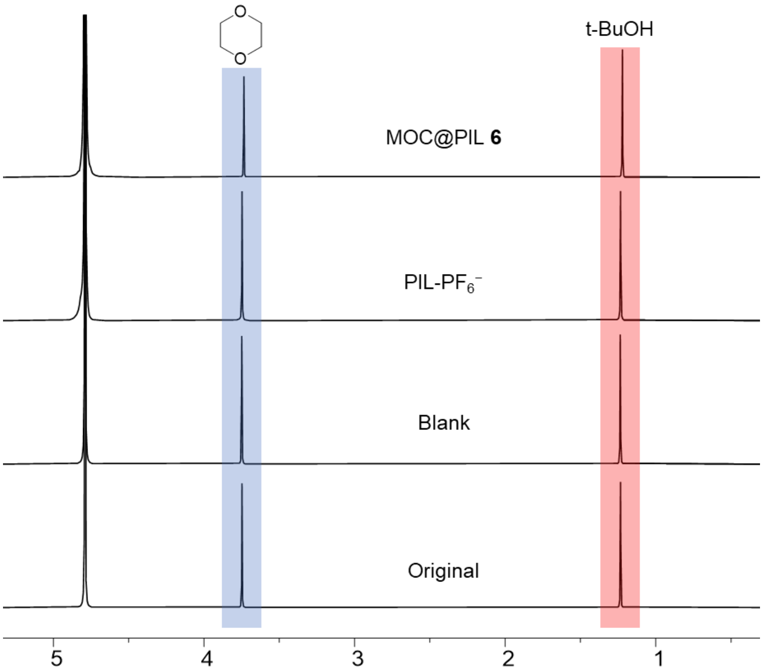

Supplementary Figure 58. ^{1}H NMR (D_2O, 400 MHz, 298 K, number of scans = 64) spectra of the polluted water before and after adsorption of 1,4-dioxane. The initial concentration of 1,4-dioxane was 5 mM. The concentration of 1,4-dioxane was reduced to 3.75 mM after adsorption with MOC@PIL **6**.

Adsorption of cyclohexene

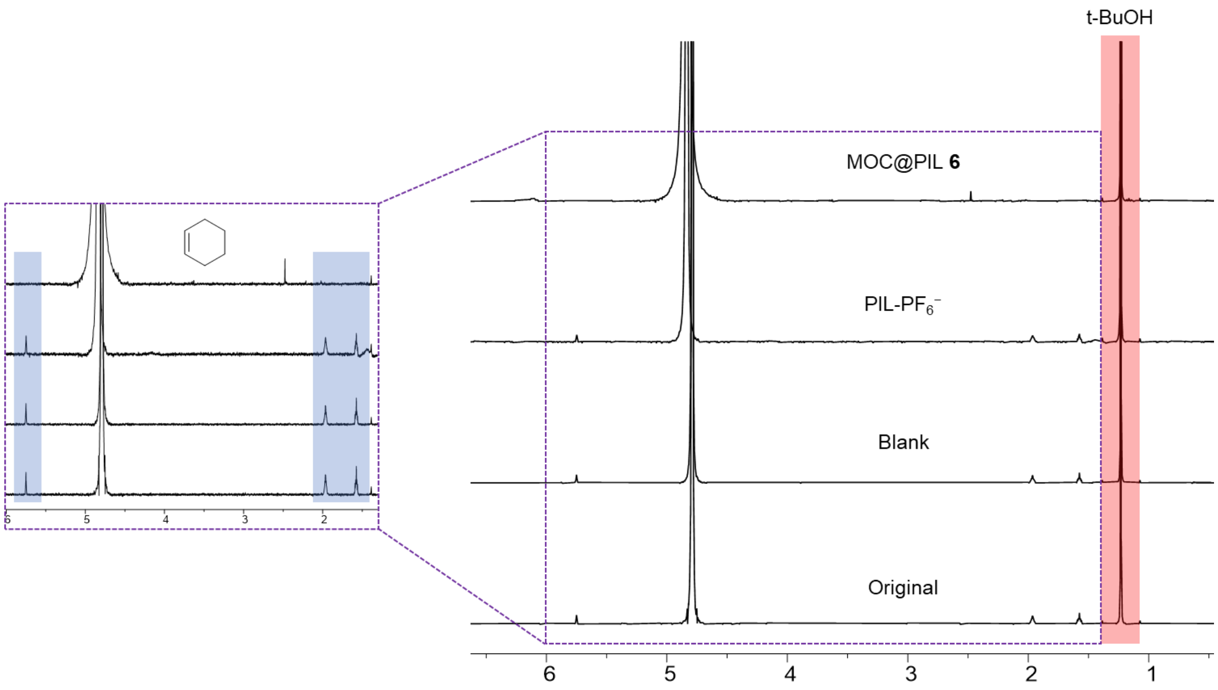

Supplementary Figure 59. ^{1}H NMR (D_2O, 400 MHz, 298 K, number of scans = 64) spectra of the polluted water before and after adsorption of cyclohexene. The initial concentration of cyclohexene was 5 mM.

Adsorption of cyclohexane

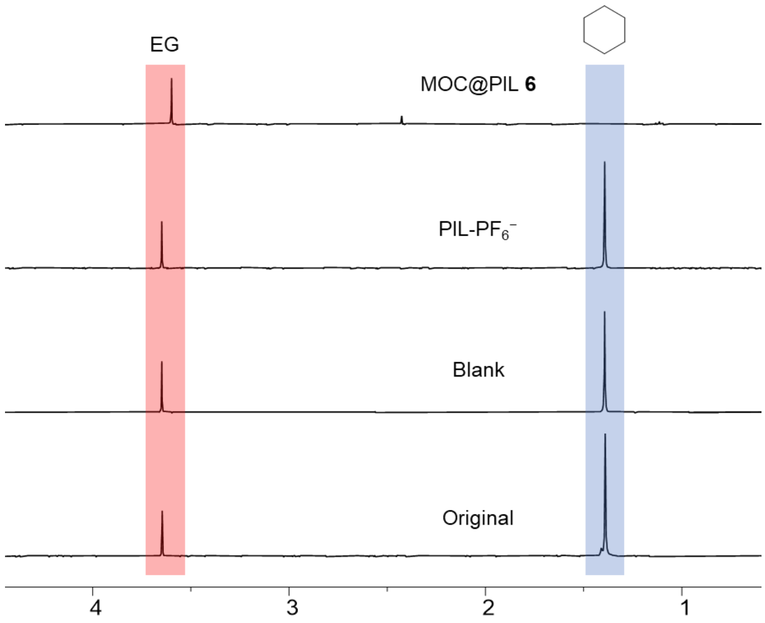

Supplementary Figure 60. ^{1}H NMR (D_2O, 400 MHz, 298 K, number of scans = 64) spectra of the polluted water before and after adsorption of cyclohexane. The initial concentration of cyclohexane was 5 mM.

Adsorption of norbornadiene

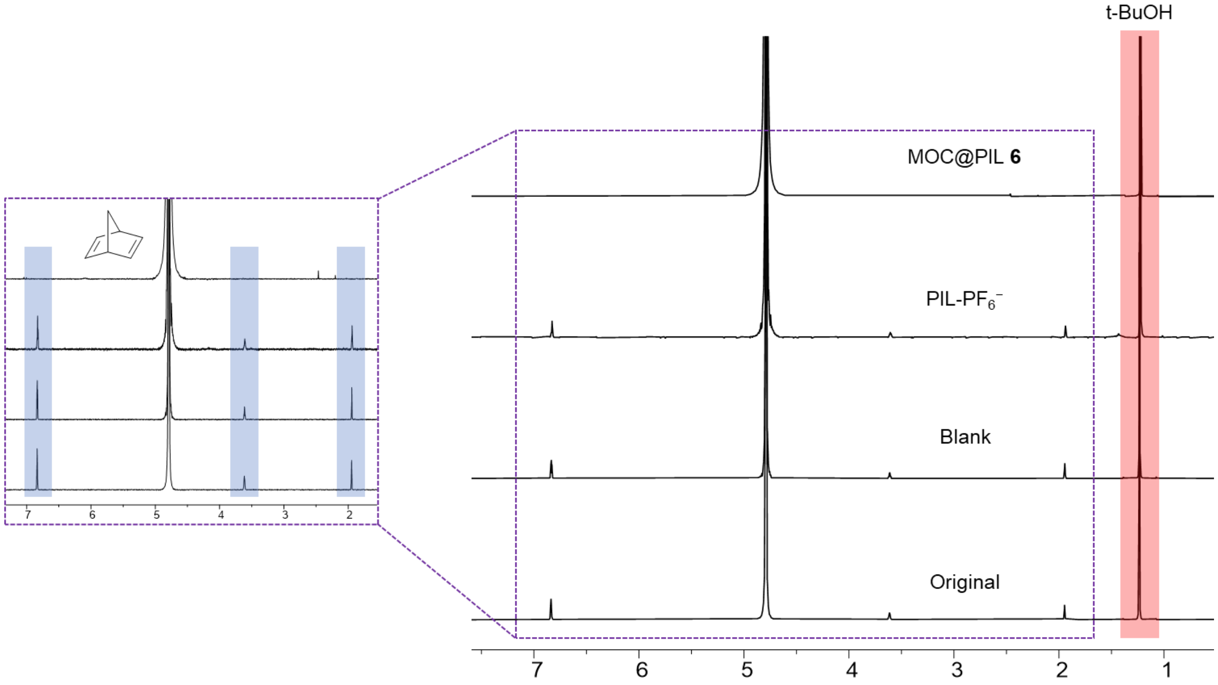

Supplementary Figure 61. ^{1}H NMR (D_2O, 400 MHz, 298 K, number of scans = 64) spectra of the polluted water before and after adsorption of norbornadiene. The initial concentration of norbornadiene was 5 mM.

Adsorption of norbornene

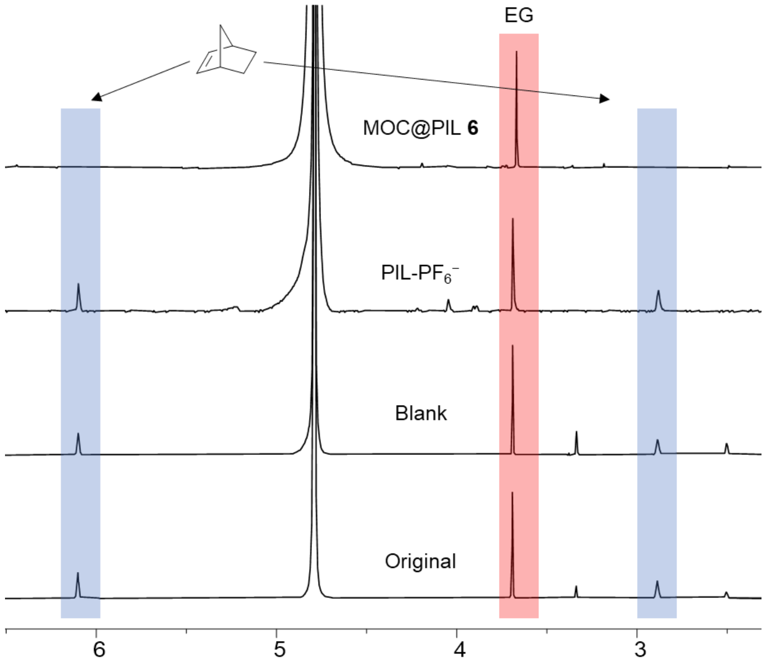

Supplementary Figure 62. ^{1}H NMR (D_2O, 400 MHz, 298 K, number of scans = 64) spectra of the polluted water before and after adsorption of norbornene. The initial concentration of norbornene was 1.34 mM.

Adsorption of norbornane

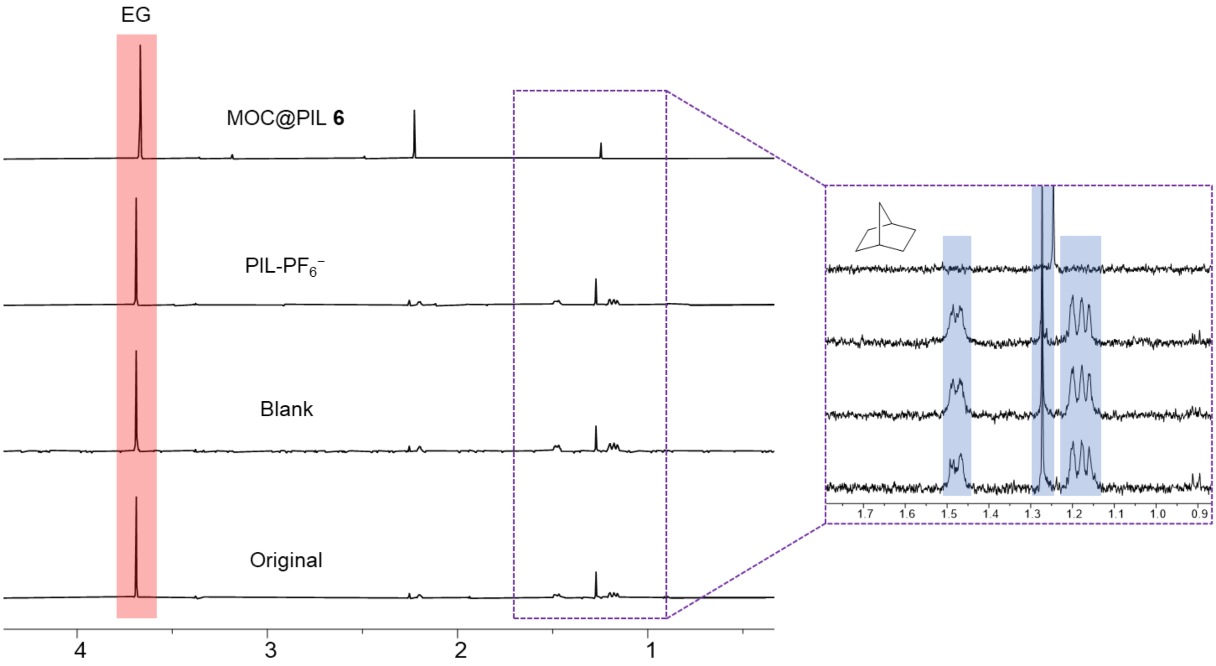

Supplementary Figure 63. ^{1}H NMR (D_2O, 400 MHz, 298 K, number of scans = 64) spectra of the polluted water before and after adsorption of norbornane. The initial concentration of norbornane was 1.46 mM.

Adsorption of 7-oxabicycloheptane

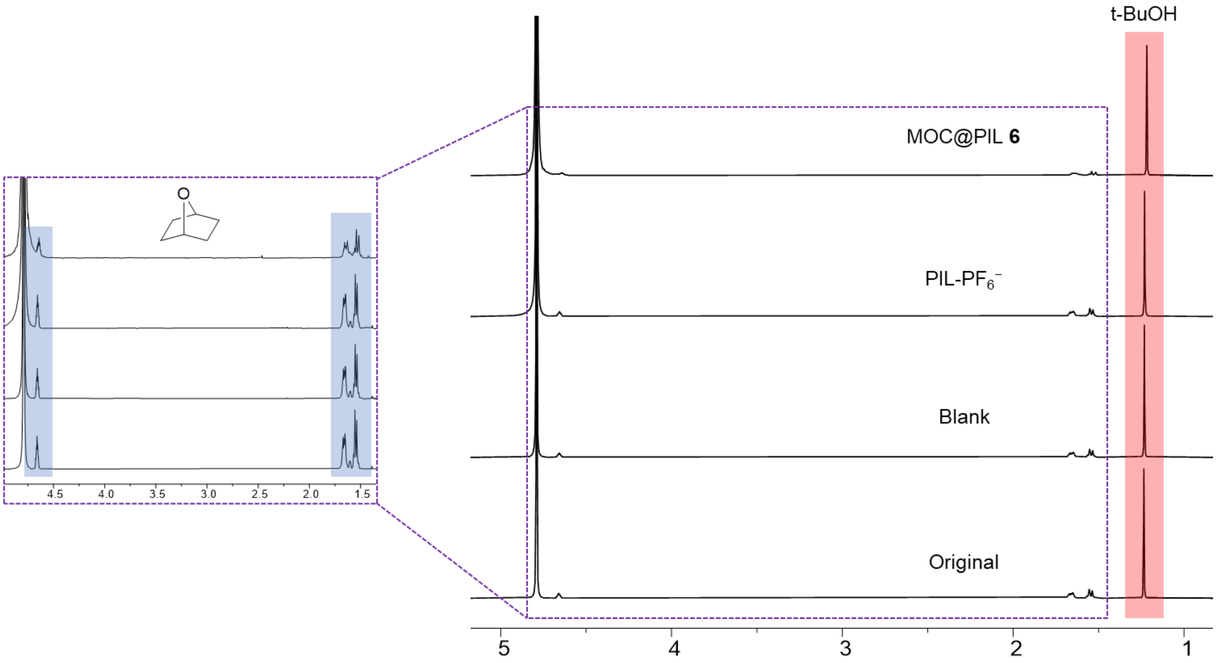

Supplementary Figure 64. ^{1}H NMR (D_2O, 400 MHz, 298 K, number of scans = 64) spectra of the polluted water before and after adsorption of 7-oxabicycloheptane. The initial concentration of 7-oxabicycloheptane was 5 mM. The concentration of 7-oxabicycloheptane was reduced to 3.33 mM after adsorption with MOC@PIL **6**.

For the removal of CH_2Cl_2, $CHCl_3$, benzene, 1,4-cyclohexadiene, cyclohexene, cyclohexane, norbornadiene, norbornene, and norbornane from water, almost no proton signal of guests could be observed after adsorption by MOC@PIL **6**. In order to accurately calculate the removal efficiency, the sensitivity of the ^{1}H NMR technique should be known. Benzene samples at various concentrations (22.9, 10, 1, 0.1, and 0.05 mM) in D_2O were thus measured by the same piece of NMR equipment with the same number of scans (64 scans) at 298 K for each sample. As shown in Supplementary Figure 65, the signal of 0.05 mM benzene, equivalent to “3 mM C-H”, in D_2O was still visible on the ^{1}H NMR spectrum. Based upon the probed sensitivity, the removal rates of these organic pollutants were calculated and tabulated in Supplementary Table 5. It should be noted that as no signal of guests could be observed after adsorption by MOC@PIL **6** on the ^{1}H NMR spectra, the pollutant removal data were underestimated and only the lower limits are provided.

For the removal of 1,4-dioxane and 7-oxabicycloheptane from water, removal rates were calculated directly based upon the relative intensities of the signals of the guests and the *tert*-butanol internal standard.

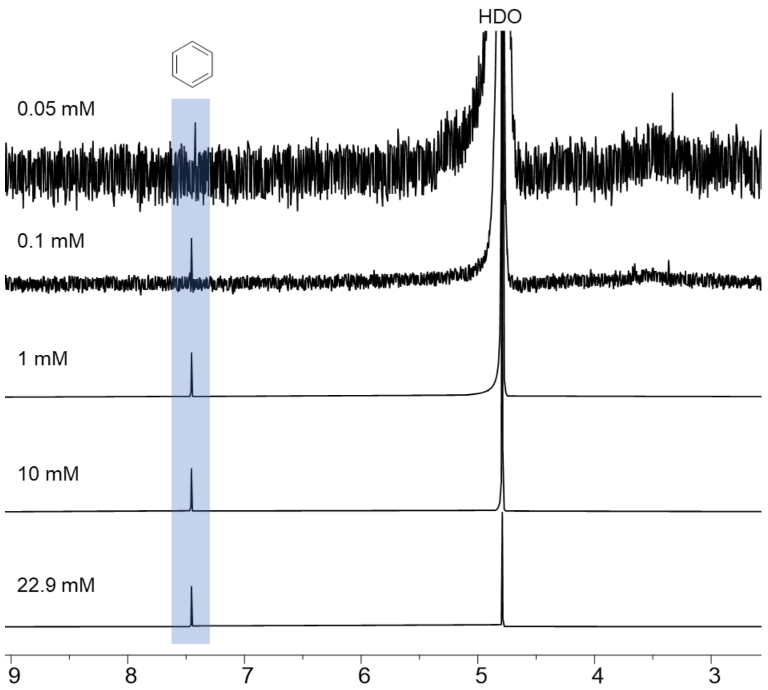

Supplementary Figure 65. ^{1}H NMR (D_2O, 400 MHz, 298 K, number of scans = 64) spectra of benzene at various concentrations (22.9 mM, 10 mM, 1 mM, 0.1 mM and 0.05 mM) in D_2O.

Supplementary Table 5. Removal rates (%) of the organic pollutants from D_2O by adding MOC@PIL **6**.

guest	removal rate (%)
CH_2Cl_2	≥ 97
$CHCl_3$	≥ 94
benzene	≥ 99
1,4-cyclohexadiene	≥ 98
1,4-dioxane	25
cyclohexene	≥ 98
cyclohexane	≥ 99
norbornadiene	≥ 98
norbornene	≥ 98
norbornane	≥ 99
7-oxabicycloheptane	45

5.2 Regeneration of MOC@PILs

Regeneration of the MOC@PIL adsorbent could be achieved through the use of acetone. The addition of acetone (5 mL) to the swollen (guest⊂MOC)@PIL resulted in the deswelling of the gel along with the extraction of the guest into the acetone phase. The release of the bound contaminant into acetone could be verified by the detection with GC analysis (Supplementary Figure 66). The ^{1}H NMR spectrum of the anionic cage in D_2O released from regenerated MOC@PIL **6** indicated the presence of empty cage alone (Supplementary Figure 67), demonstrating the complete release of the guest from the immobilized cage. The regenerated adsorbent MOC@PIL **6** was reused for adsorption of benzene five times following the procedure described at the beginning of Section 5 (Supplementary Figure 68).

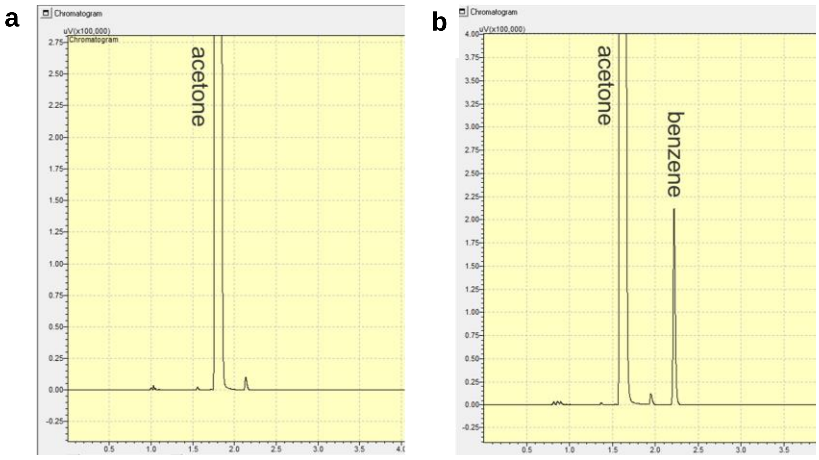

Supplementary Figure 66. GC spectra of (a) pure acetone and (b) the acetone phase used for the treatment of (benzene⊂MOC)@PIL **6**, in which benzene has been detected.

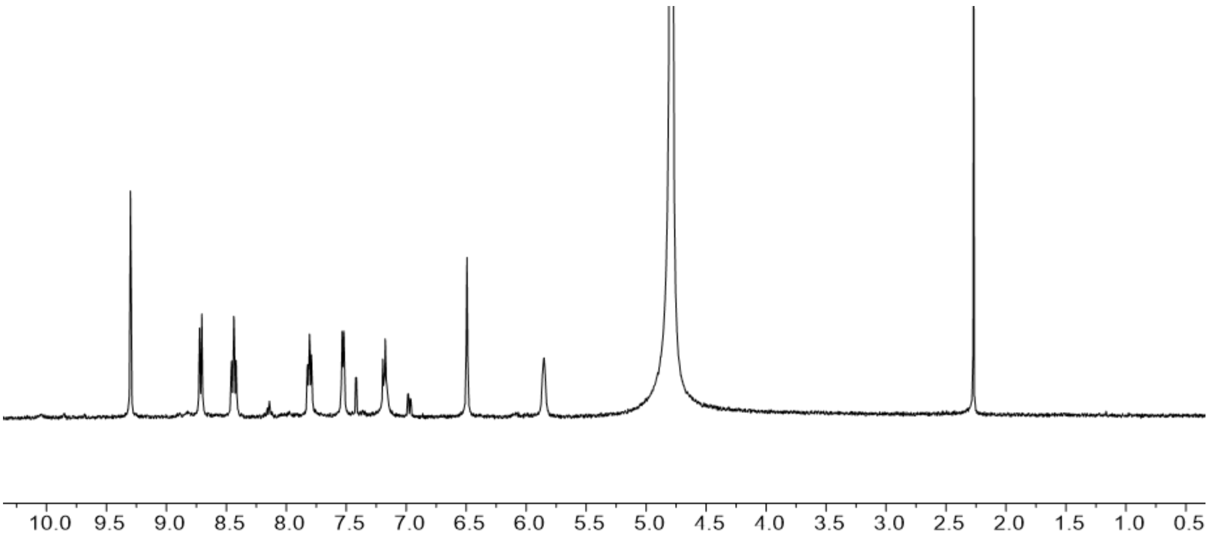

Supplementary Figure 67. ^{1}H NMR (D_2O, 400 MHz, 298 K) spectrum of the cage released from regenerated MOC@PIL **6** by adding $NaNO_3$. Only empty cage is observed with no appearance of peaks of benzene⊂MOC.

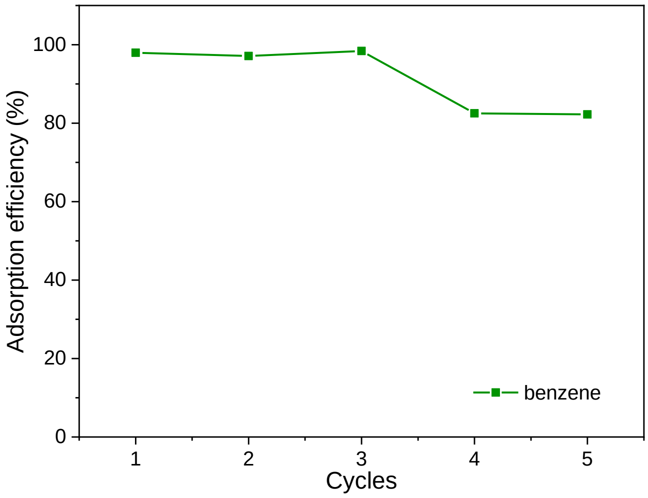

Supplementary Figure 68. Efficiency of benzene adsorption with the use of the recycled MOC@PIL **6**.

6. Purification of organic chemicals

MOC@PIL **6** was first applied as the adsorbent for the purification of toluene (97.9%, containing 2.1% benzene, wt%). Guest binding studies indicated the ability of MOC@PILs for the binding of benzene, while toluene was not a preferred guest for the immobilized cage. Swollen MOC@PIL **6** (250 mg, in 1.5 mL D_2O) was thus thoroughly mixed with 100 μL of contaminated toluene, and the mixture was stirred at rt for 15 h. The organic phase was separated and the purity of toluene was quantified by GC analysis. For comparison, the control experiment was also carried out under the same conditions except the addition of MOC@PIL **6**.

In contrast to the binding of cyclohexane that required heating to facilitate equilibration, the binding of benzene and 1,4-cyclohexadiene with MOC@PILs were much faster and could be achieved at rt. MOC@PIL **6** was thus used for the purification of cyclohexane (98.1%, containing 0.9% benzene and 1.0% 1,4-cyclohexadiene) following the same procedure used for the purification of toluene.

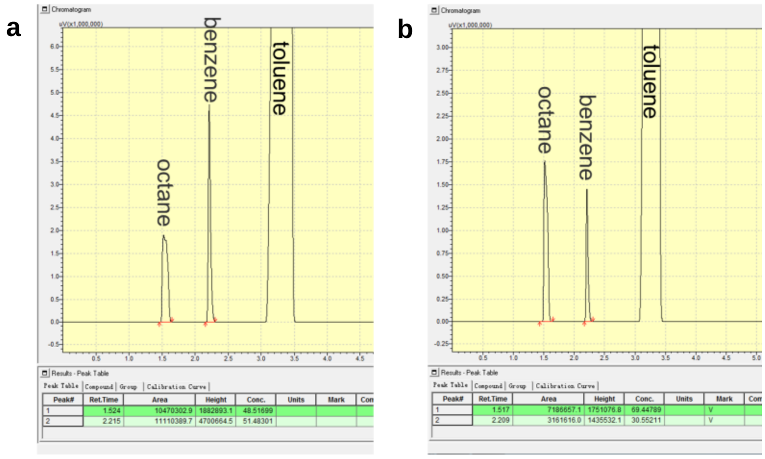

Supplementary Figure 69. GC spectra of the final toluene solution (a) in the control experiment and (b) purified with MOC@PIL **6**. Octane was used as the internal standard.

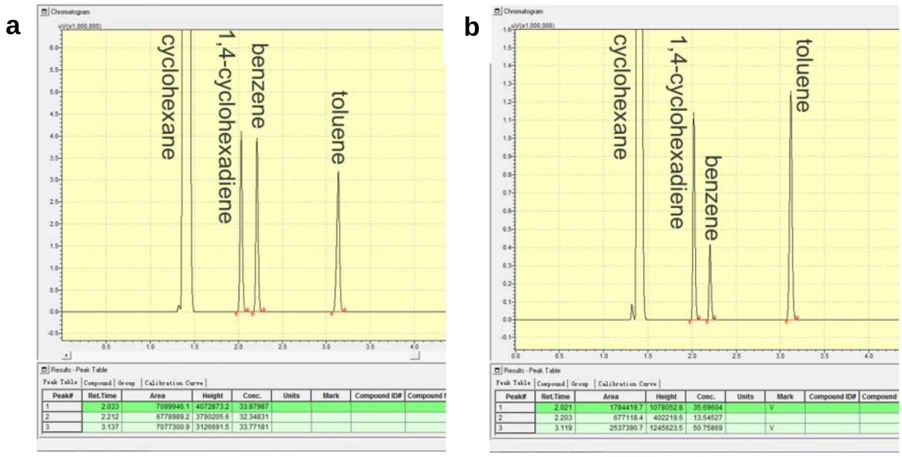

Supplementary Figure 70. GC spectra of the final cyclohexane solution (a) in the control experiment and (b) purified with MOC@PIL **6**. Toluene was used as the internal standard.

7. Supplementary Notes

The geometry optimized structures of guests and new guest⊂$Fe^{II}_4L_6$ complexes were modeled at the PM7[7] level of theory using the MOPAC program (version 22.0.5).[8] The starting $Fe^{II}_4L_6$ cage structure was a previously reported crystal structure (CCDC 784594).

Asphericity Ω_A of guest molecules was calculated from their principal moments of inertia (I_A, I_B, and I_C) following the equation below:

$$\Omega_A = \frac{(I_A - I_B)^2 + (I_A - I_C)^2 + (I_B - I_C)^2}{2(I_A + I_B + I_C)^2} \quad (4)$$

Moments of inertia of the molecules were calculated with the program "Moments of intertia" (v1.3.1) downloaded from https://github.com/solamir/moments_of_inertia (05-08-2023).[9]

Volume calculations of guests and the cage cavity were performed using the MoloVol program (version 1.1.0)[10] with the following parameters:

Crystal unit cell analysis: No
Probe mode: one probe
Probe radius: 1.2 Å
Grid resolution: 0.1 Å
Optimization depth: 4
Elements radii:
S : 1.89 Å
O : 1.50 Å
N : 1.66 Å
H : 1.20 Å
Fe : 2.44 Å
Cl : 1.82 Å
C : 1.77 Å

8. Supplementary References

[1] P. Mal, D. Schultz, K. Beyeh, K. Rissanen, J. R. Nitschke, *Angew. Chem.* **2008**, *47*, 8297-8301.

[2] Y. Zhang, B. Chen, Y. Zhang, L. Qin, B. Liu, B. Ni, G. Gao, *Green Chem.* **2018**, *20*, 1594-1601.

[3] a) O. Chantarasriwong, D. O. Jang, W. Chavasiri, *Tetrahedron Lett.* **2006**, *47*, 7489-7492; b) H. Zhou, J. Song, X. Kang, J. Hu, Y. Yang, H. Fan, Q. Meng, B. Han, *RSC Adv.* **2015**, *5*, 15267-15273.

[4] a) X. Tang, C. Qi, H. He, H. Jiang, Y. Ren, G. Yuan, *Adv. Synth. Cat.* **2013**, *355*, 2019-2028; b) D. Fu, Y. Sun, F. Zhang, Z. Sun, W. Li, L. Wang, Z. Wang, A. Pan, J. Xu, X. Wu, *Chem. Eng. J.* **2022**, *431*.

[5] M. M. Smulders, S. Zarra, J. R. Nitschke, *J. Am. Chem. Soc.* **2013**, *135*, 7039-7046.

[6] M. H. Abraham, J. Le, *J. Pharm. Sci.* **1999**, *88*, 868-880.

[7] J. J. P. Stewart, *J. Mol. Mod.* **2013**, *19*, 1–32.

[8] J. J. P. Stewart A. Klamt, W. Thiel, D. Danovich, G. B. Rocha, R. L. Gieseking, J. E. Moussa, H. A. Kurtz, P. Korambath, K. M. Merz, B. Wang. MOPAC (22.0.5). *Zenodo* **2022**. https://doi.org/10.5281/zenodo.7453815

[9] M. Korveda. Program Moment of intertia. *Mendeley Data* **2020**, Version 1. DOI: https://doi.org/10.17632/skj4999wr6.1

[10] J. B. Maglic, R. Lavendomme, *J. Appl. Cryst.* **2022**, *55*, 1033–1044.
